# Extensive diversity of Rickettsiales bacteria in two species of ticks from China and the evolution of the Rickettsiales

**DOI:** 10.1186/s12862-014-0167-2

**Published:** 2014-07-30

**Authors:** Yan-Jun Kang, Xiu-Nian Diao, Gao-Yu Zhao, Ming-Hui Chen, Yanwen Xiong, Mang Shi, Wei-Ming Fu, Yu-Jiang Guo, Bao Pan, Xiao-Ping Chen, Edward C Holmes, Joseph J Gillespie, Stephen J Dumler, Yong-Zhen Zhang

**Affiliations:** 1 Department of Zoonoses, State Key Laboratory for Infectious Disease Prevention and Control, National Institute for Communicable Disease Control and Prevention, Chinese Center for Disease Control and Prevention, Changping Liuzi 5, Beijing 102206, China; 2 Center for Diagnosis and Treatment of Infectious Diseases, Hangzhou, China; 3 Veterinary Station, Jiulingtuan of Nongwushi, Bole, Xinjiang Uygur Autonomous Region, China; 4 Veterinary Station, Emin, Nongjiushi, Xinjiang Uygur Autonomous Region, China; 5 Marie Bashir Institute for Infectious Diseases and Biosecurity, Charles Perkins Centre, School of Biological Sciences and Sydney Medical School, The University of Sydney, Sydney, Australia; 6 Departments of Pathology and Microbiology & Immunology, University of Maryland School of Medicine, Baltimore, MD, USA

**Keywords:** Co-divergence, Evolution, Phylogeny, Rickettsiales bacteria, Ticks, Vectors

## Abstract

**Background:**

Bacteria of the order Rickettsiales (*Alphaproteobacteria*) are obligate intracellular parasites that infect species from virtually every major eukaryotic lineage. Several rickettsial genera harbor species that are significant emerging and re-emerging pathogens of humans. As species of Rickettsiales are associated with an extremely diverse host range, a better understanding of the historical associations between these bacteria and their hosts will provide important information on their evolutionary trajectories and, particularly, their potential emergence as pathogens.

**Results:**

Nine species of Rickettsiales (two in the genus *Rickettsia*, three in the genus *Anaplasma*, and four in the genus *Ehrlichia*) were identified in two species of hard ticks (*Dermacentor nuttalli* and *Hyalomma asiaticum*) from two geographic regions in Xinjiang through genetic analyses of 16S rRNA, *gltA*, and *groEL* gene sequences. Notably, two lineages of *Ehrlichia* and one lineage of *Anaplasma* were distinct from any known Rickettsiales, suggesting the presence of potentially novel species in ticks in Xinjiang. Our phylogenetic analyses revealed some topological differences between the phylogenies of the bacteria and their vectors, which led us to marginally reject a model of exclusive bacteria-vector co-divergence.

**Conclusions:**

Ticks are an important natural reservoir of many diverse species of Rickettsiales. In this work, we identified a single tick species that harbors multiple species of Rickettsiales, and uncovered extensive genetic diversity of these bacteria in two tick species from Xinjiang. Both bacteria-vector co-divergence and cross-species transmission appear to have played important roles in Rickettsiales evolution.

## Background

Bacteria of the order Rickettsiales are obligate intracellular parasites of eukaryotes. While some symbionts are known (for example, many *Wolbachia* species), most described species of Rickettsiales are best known as human pathogens that cause several diseases, including rickettsioses, anaplasmosis, and ehrichiosis [1]. Historically, rickettsial agents have been important causes of human morbidity and mortality, including *R. prowazekii* that caused several million deaths in the USSR [2], and it is estimated that *Orientia tsutsugamushi* is currently responsible for approximately one million cases of scrub typhus per year [2],[3]. The discovery of new pathogenic species or their associated diseases has attracted attention to the Rickettsiales as pathogens [4]-[8]. As their arthropod vectors often live at high densities and in close proximity to domestic animals and humans, Rickettsiales will continue to pose a risk for transmission to humans. Hence, the identification and characterization of novel Rickettsiales is of importance for both animal and human health.

The number of novel Rickettsiales associated with protists, arthropods, and mammals has increased rapidly through the application of molecular detection and phylogenetics [4]-[6],[9],[10]. Remarkably, analysis of the *Trichoplax adhaerens* genome also reveals novel species in the order Rickettsiales (for example, [11]). At present, this order contains three established families (*Rickettsiaceae*, *Anaplasmataceae,* and *Holosporaceae*) and one proposed family (*Candidatus* Midichloriaceae) [8],[11]-[14]. Additionally, some unclassified species warrant further attention to determine their phylogenetic and systematic positions [8],[11]. The intra- and inter-species genetic diversity and evolutionary relationships within genera of Rickettsiales bacteria have been characterized using 16S rRNA gene (*rrs*) sequences, especially in the case of those bacteria causing animal and human disease [5],[6],[9],[14],[15]. However, relatively little is known about their potential for cross-species transmission and emergence.

Compared with other zoonotic or vector-borne bacteria, Rickettsiales are associated with a more extremely diverse host range, including protists, hydra, annelids, arthropods, vertebrates, and even plants [5],[8],[15],[16]. While some Rickettsiales are specific to particular vectors and hosts [16],[17], others experience host-switching or regularly cycle between different hosts, typically a mammal (e.g. rodents, cattle and humans) and a blood-feeding arthropod (e.g. fleas, mites and ticks) [5],[16],[17]. However, the evolutionary associations between Rickettsiales and their hosts are not well understood [6],[16],[18],[19]. In particular, it is unclear whether Rickettsiales most often evolve by long-term bacteria-host co-divergence or cross-species transmission [20],[21]. As most emerging infectious diseases in humans are caused by spillover from animal hosts or vectors, a better understanding of the evolutionary relationships among *Rickettsiales* bacteria could provide important information on the likelihood of their emergence as agents of disease.

Xinjiang (one of five autonomous regions of China) is located in the northwestern part of China, and borders Russia, Mongolia, Kazakhstan, Kyrgyzstan, Tajikistan, Afghanistan, Pakistan and India (Additional file 1: Figure S1) and is one of the nation’s major grazing areas. Several important tick-borne diseases are endemic in Xinjiang [22]. The main aim of this study was to explore the diversity of Rickettsiales in Xinjiang, China, where their presence has only previously been shown by serological data [23],[24]. Accordingly, we screened ticks and identified bacteria by sequencing and analyzing three genes; *rrs*, citrate synthase (*gltA*), and heat shock protein (*groEL*). With these data in hand we explored key aspects of Rickettsiales biodiversity and evolution.

## Results

### Collection of ticks and detection of Rickettsiales bacterial DNA

In the spring of 2011, a total of 2062 adult ticks were collected from domestic animals (sheep and cattle) and grasslands in the border areas of the Bole and Tacheng regions of Xinjiang Uygur Autonomous Region, China (Additional file 1: Figure S1). The numbers, species, and geographic distributions of the adult ticks collected are shown in Table 1. After morphological examination and sequence analysis of mitochondrial 18S and 12S rDNA sequences as described previously [25], only *Dermacentor nuttalli* and *Hyalomma asiaticum* were found in Xinjiang.

**Table 1:** **Detection of** ***Rickettsiales*** **bacteria from pooled tick samples**

**Tick Species**	**Origin**	**No. of PCR-positive tick pools/total no. of ticks collected**		
		**Bole**	**Tacheng**	**Subtotal**
*Hyalomma asiaticum*	Grassland	19/1023	-	19/1023
	Cattle	5/303	-	5/303
	Sheep or goats	11/285	-	11/285
*Dermacentor nuttalli*	Sheep or goats	0/15	37/236	37/251
**Total**		35/1626	37/236	72/1862

A total of 388 tick pools (1862 ticks) were investigated in this study, 314 of which were from Bole and 74 from Tacheng. PCR was performed to detect Rickettsiales DNA based on *rrs*. PCR products of the expected size were amplified from 50 tick pools from Bole and 37 from Tacheng. Genetic analyses of these sequences indicated that all products belonged to Rickettisales (see below).

### Genetic analysis of bacterial DNA sequences

The *rrs*, *gltA*, and *groEL* gene sequences amplified from the Rickettsiales DNA-positive tick-pool samples were sequenced (sequences are described in detail in Additional file 2: Table S1). Genetic analyses indicated that all sequences recovered from ticks from Xinjiang shared strong similarities with those from species of *Anaplasma*, *Ehrlichia*, and *Rickettsia* (with percentages greater than 97%, 97.9% and 98.8%, respectively, in the *rrs* gene), and hence within the standard reference values used for assignments to these genera (i.e. above 96%, 97.6% and 97.2% with the genus *Anaplasma*, *Ehrlichia*, and *Rickettsia* SFG group, respectively) [26]. Hence, these bacterial groups circulate in *Dermacentor* and *Hyalomma* ticks in Xinjiang (Additional file 2: Table S1). A PCR based on individual tick samples confirmed the likelihood that in each case the three genes of each identified species from tick pool are from a single bacterial species.

### Phylogenetic relationships between newly identified and known Rickettsiales

**Figure 1:**
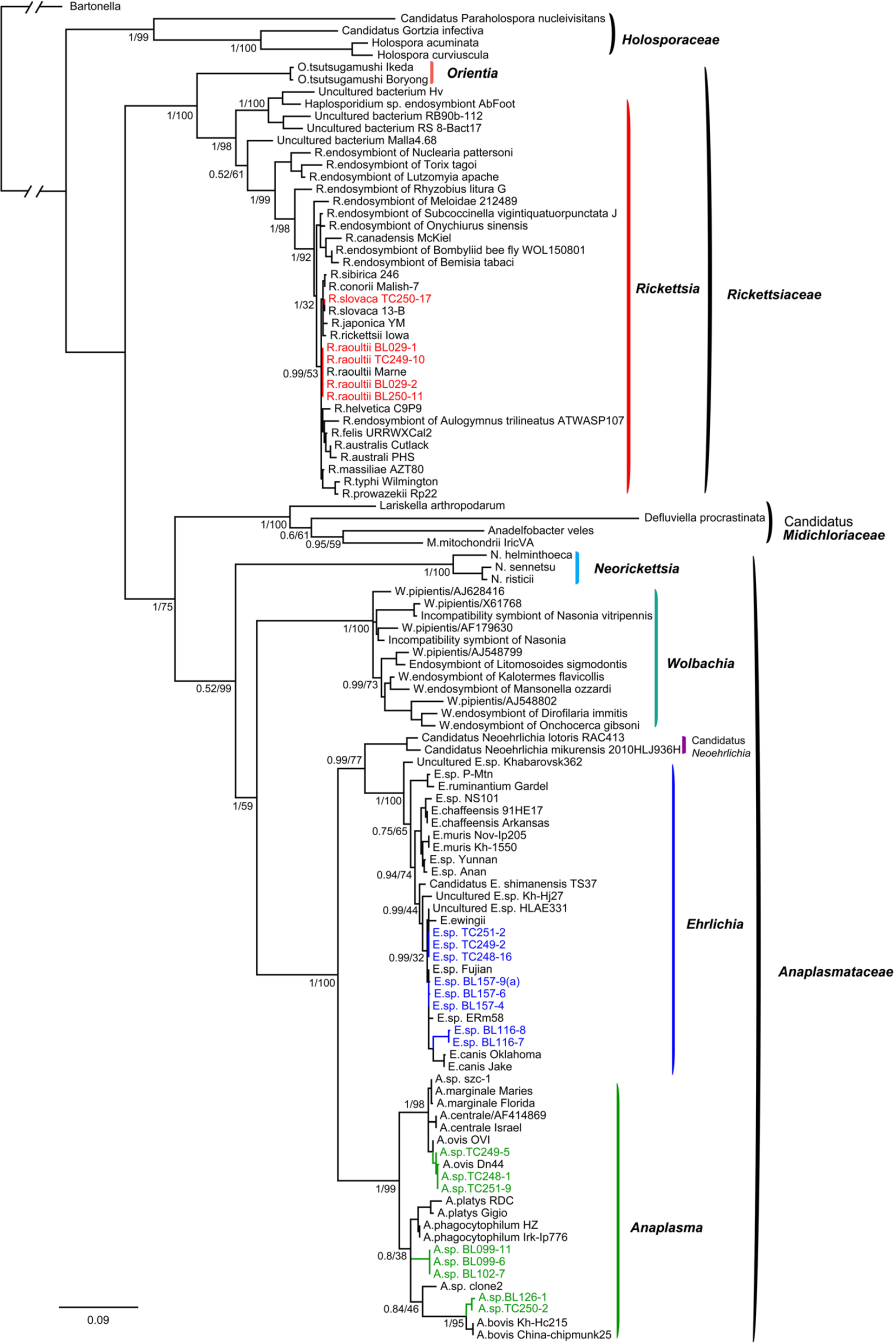
**Phylogenetic trees based on partial Rickettsiales*rrs*sequences using Bayesian (MrBayes and ML (PhyML methods.** Numbers at each branch indicate posterior probabilities for the Bayesian (left and bootstrap values for the ML (right trees. The ML tree is shown here.

To determine the phylogenetic relationships among the Rickettsiales bacteria identified here and those described previously, we estimated phylogenetic trees based on the *rrs*, *gltA*, and *groEL* genes using ML and Bayesian methods, all of which produced similar topologies. In agreement with previous studies [8],[11], all *Rickettsiales* bacteria including those identified in this study were classified into four well-supported monophyletic groups in the *rrs* trees (Figure 1), corresponding to the families *Holosporaceae, Rickettsiaceae*, *Candidatus* Midichloriaceae, and *Anaplasmataceae*. The family *Rickettsiaceae* comprises two genera – *Orientia* and *Rickettsia* – while the family *Anaplasmataceae* contains the genera *Neorickettsia*, *Wolbachia*, *Ehrlichia*, *Anaplasma*, and *Candidatus* genus Neoehrlichia. As the *gltA* gene of *O. tsutsugamushi* strains is a pseudogene, the ML and Bayesian trees based on *gltA* gene sequences did not include *O. tsutsugamushi*, although this did not change the topological positions of the other taxa.

**Figure 2:**
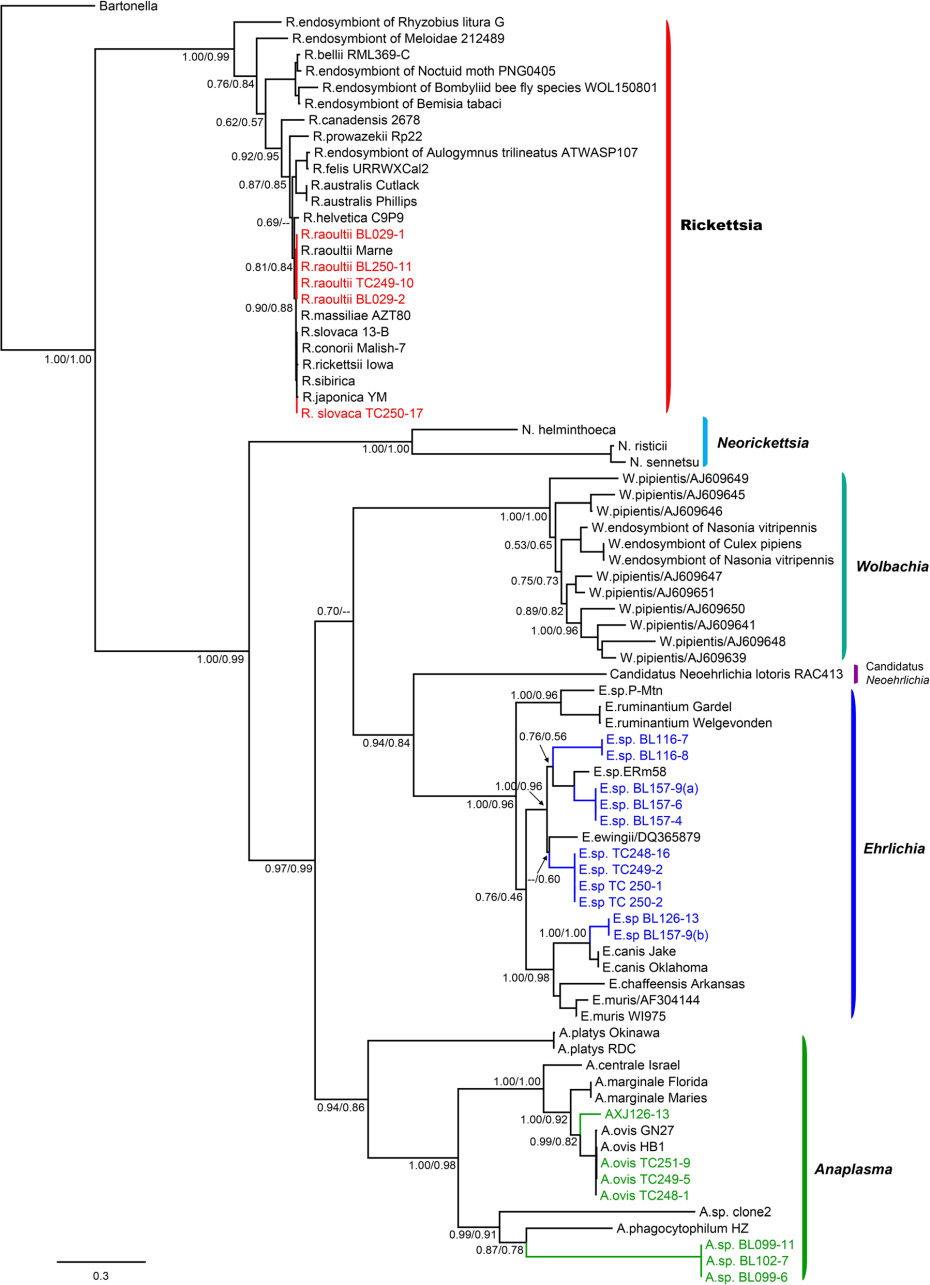
**Phylogenetic trees based on the parital coding region of citrate synthase gene (*gltA*of order Rickettsiales bacteria using the Bayesian and ML methods.** The figure description follows that in Figure 1.

**Figure 3:**
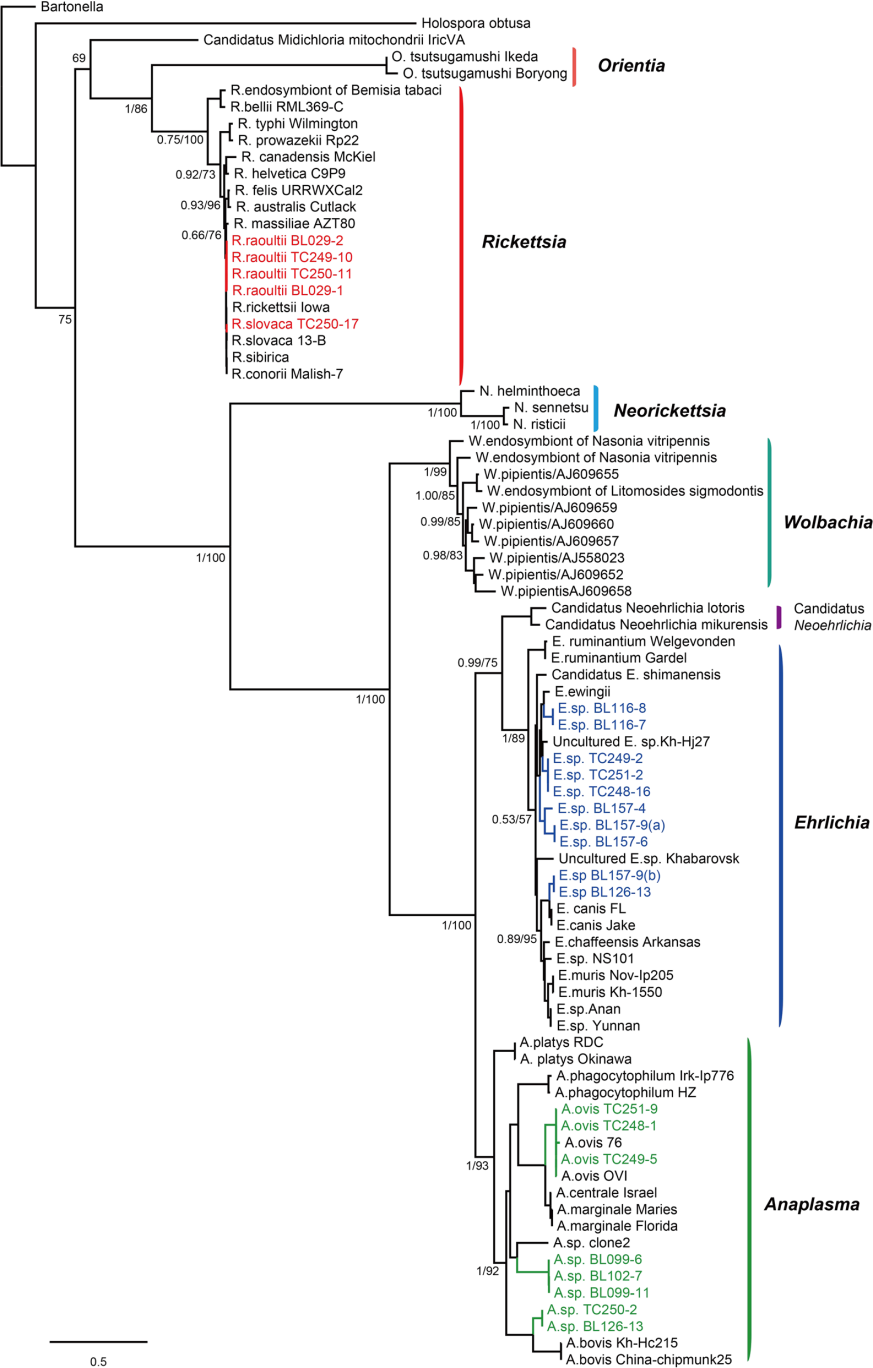
**Phylogenetic trees based on the parital coding region of heat shock protein gene (*groEL*of order Rickettsiales bacteria using the Bayesian and ML methods.** The figure description follows that in Figure 1.

Within the genus *Rickettsia*, the bacteria (*R. raoultii* BL029-1, *R. raoultii* BL029-2, *R. raoultii* TC249-10, and *R. raoultii* TC250-11) identified in ticks from the Bole and Tacheng regions were closely related to the species *R. raoultii* carried by *Dermacentor* spp*.* and *R. pumilio* ticks [27] in the *rrs* tree (Figure 1, Additional file 3: Figure S2a), while the sequences (*R. slovaca* TC250-17) recovered from ticks from the Tacheng region had a closer evolutionary relationship with *R. slovaca* isolated from *Dermacentor* spp. [28]. Hence, at least two species of *Rickettsia* circulate in ticks from the Bole and Tacheng regions of Xinjiang. Similar clustering patterns were observed in the trees inferred from *groEL* and *glt*A sequences (Figures 2, 3, and Additional file 3: Figure S2bc).

Within the genus *Ehrlichia*, the sequences (*Ehrlichia* sp. TC251-2, *Ehrlichia* sp. TC249-2, and *Ehrlichia* sp. TC248-16) recovered from *D. nuttalli* ticks from Tacheng clustered in the *rrs* tree with *E. ewingii* carried by *A. americanum* and *D. variabilis* ticks [29],[30] (Figure 1, Additional file 3: Figure S2d). These sequences also clustered together in both *gltA* and *groEL* trees (Figures 2, 3, and Additional file 3: Figure S2ef), but were distinct from *E. ewingii*, suggesting that they represent a potential new species of *Ehrlichia* in ticks from Tacheng region. The bacterial sequences (*Ehrlichia* sp. BL157-9, *Ehrlichia* sp. BL157-4, and *Ehrlichia* sp. BL157-6) identified in *H. asiaticum* ticks from Bole clustered in *rrs* trees together with the *Ehrlichia*. sp. ERm58 sequences identified in *Rhipicephalus muhsamae* ticks [31] and *Ehrlichia*. sp. Fujian identified in *R. microplus* ticks from China [32]. However, the evolutionary relationships of these five bacterial sequences were not well resolved in the *rrs* trees. Interestingly, they shared a relatively close evolutionary relationship with *Ehrlichia*. sp. ERm58 in the *gltA* tree, but were a distinct lineage in the *groEL* tree, suggesting the possible presence of a new variant of *Ehrlichia*. sp. ERm58 in ticks from Bole. The bacterial sequences (*Ehrlichia* sp. BL116-7 and *Ehrlichia* sp. BL116-8) recovered from *H. asiaticum* ticks from Bole formed a distinct lineage in the *rrs* tree, and showed a relatively close relationship with *E. canis* primarily transmitted by *R. sanguineus* and *D. variabilis*[33],[34]. They also formed a distinct lineage in both *gltA* and *groEL* trees, possibly indicative of a new species.

Within the genus *Anaplasma*, the bacterial sequences (*A. ovis* TC249-5, *A. ovis* TC248-1, and *A. ovis* TC251-9) recovered from *D. nuttalli* ticks from Tacheng were closely related to *A. ovis* bacteria in *Dermacentor* spp. and *Rhipicephalus* spp. [9] in the *rrs*, *gltA*, and *groEL* trees (Figures 1, 2, 3, and Additional file 3: S2ghi). In the *rrs* and *groEL* trees the bacterial sequences (*Anaplasma* sp. BL126-13 and *Anaplasma* sp. TC250-2) identified in *H. asiaticum* ticks from Bole and *D. nuttalli* ticks from Tacheng clustered together and showed a close relationship with the species *A. bovis*, which is predominantly vectored by *Amblyomma spp., Rhipicephalus spp.,* and *Hyalomma spp*. [9]. As *A. bovis gltA* sequences are not available, the sequence (*Anaplasma* sp. BL126-13) formed a distinct lineage. Remarkably, the sequences (*Anaplasma* sp.BL102-7, *Anaplasma* sp.BL099-6, and *Anaplasma* sp.BL11) recovered from *H. asiaticum* ticks from Bole were divergent from any known *Anaplasma* bacteria (percentage similarity > 1.6% for *rrs*, > 43.6% for *gltA*, and > 23.2% for *groEL*). They formed a distinct lineage in all three phylogenetic trees, suggesting the presence of a new *Anaplasma* species in ticks. Finally, it was notable that different clustering patterns of *Anaplasma* bacteria were observed in the trees estimated using the *groEL* and *gltA* gene sequences. Additional work is needed to determine whether these differences are due to recombination.

### Evolutionary association between Rickettsiales bacteria and their vectors

**Figure 4:**
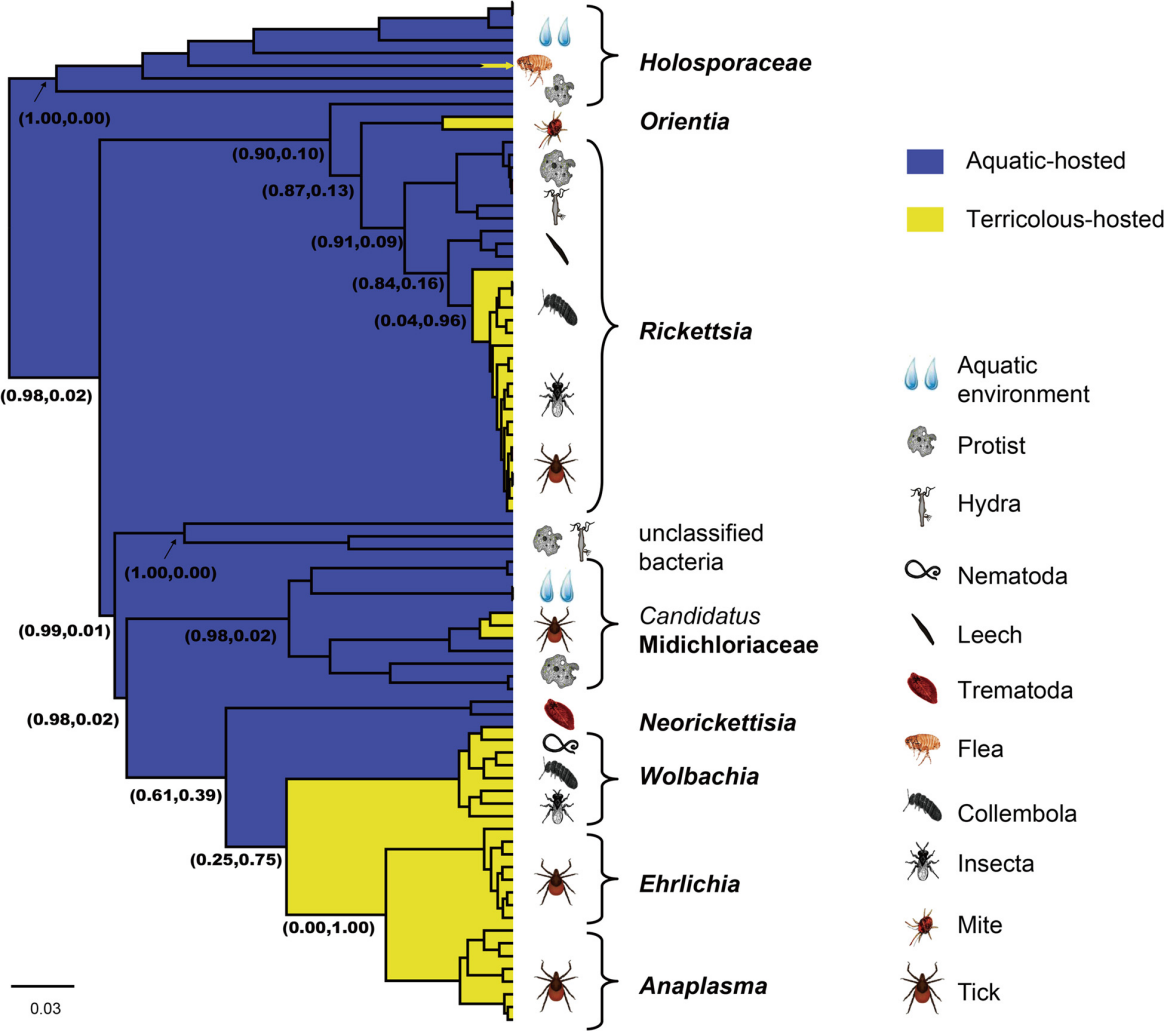
**Molecular clock (BEAST phylogeny of the order Rickettsiales based on parital 16S rRNA gene sequences.** The inferred aquatic/terrestrial traits were mapped onto the phylogeny with supporting values for each trait indicated on internal nodes.

In agreement with the recent studies [8],[11], almost all known species of the family *Holosporaceae*, which are the most divergent group in the order, are associated with protists (Figure 4 and Additional file 4: Figure S3), except one found in prairie dog flea [35]. The most divergent species within the family *Rickettsiaceae* are also predominantly associated with protists (*Diophrys appendiculata, Haplosporidium sp etc.*), and occupy the most divergent position in the phylogeny of vectors. Several exceptions include the uncharacterized species detected from *Hydra oligactis* and the leech *Torix tagoi*. All other *Rickettsia* or *Rickettsia*-like species were found in arthropods, with the majority found in ticks and a few in insects. The unclassified species, potentially a new family, are associated with protists as well as *Hydra vulgaris*[36]. Like the family *Rickettsiaceae*, bacteria from the family *Candidatus* Midichloriaceae are associated with a wide range of hosts, from protists to a variety of animals, including ticks [11],[14]. Although the bacteria of the family *Anaplasmataceae* are not found in protists, the earliest appearance is of bacteria in the genus *Neorickettsia* found within *Trematoda* or aquatic insects [37]. All known bacteria from the genera *Anaplasma* and *Ehrlichia* are found within ticks.

The evolutionary association between these species and their corresponding vectors was further evaluated with a co-phylogeny analysis. For the tick-only data sets, the null hypotheses of no co-divergence could not be rejected, although only marginally so (*P* > 0.084, Additional file 5: Table S2). Analyses based on overall data sets yielded a similar conclusion, with a P value that was closer to 0.05 (*P* = 0.064).

### Inferred ancestral habitat for the Rickettsiales

Interestingly, our phylogenetic analysis of possible ancestral character states on these data gave support to an aquatic origin for the families *Rickettsiaceae*, *Anaplasmataceae*, *Holosporaceae* and *Candidatus* Midichloriaceae with strong support values of 1.00, 0.80, 1.00 and 1.00, respectively. In addition, this analysis suggested that there were at least five independent adaptations to terrestrial animals: (1) within the family *Holosporaceae*, (2) within the genus *Rickettsia*, (3) within the currently known species of *Orientia* circulating in mites, (4) within the ancestral lineage that diverged into the genera *Wolbachia*, *Anaplasma*, and *Ehrlichia,* and (5) within the *Candidatus* Midichloriaceae, which is associated with a wide range of hosts.

## Discussion

Serological studies provided the earliest evidence for the presence of the bacteria of the genera *Anaplasma*, *Ehrlichia*, and *Rickettsia* in ticks from Xinjiang area of China [23],[24]. Since this initial work, only a small number of molecular epidemiological studies have been performed, mostly on *Rickettsiales* and limited to partial sequences of a single gene [38]. By sequencing and analyzing the bacterial sequences of complete length *rrs*, *gltA*, and *groEL* genes, we identified at least nine species of bacteria belonging to the *Rickettsia*, *Anaplasma and Ehrlichia* genera of Rickettsiales, indicating extensive genetic diversity of Rickettsiales in the two primary species of ticks in Xinjiang. Given that at least 39 species of ticks are present in Xinjiang [39], it is likely that additional tick-associated Rickettsiales circulating in this region will be discovered in the future.

Human cases of infection by Rickettsiales, leading to lymphadenopathy caused by a spotted fever group (SFG) *Rickettsia*, were documented in the 1980s in the Bole region of Xinjiang [40]. Serological analyses of the strains isolated from patients and ticks suggested that *R. sibirica* might be the etiological agent [41],[42]. In this study, phylogenetic analyses of bacterial sequences recovered from ticks indicated the presence of *R. raoultii* in Bole region and *R. slovaca* in Tacheng, species which were previously found in other regions of Xinjiang [43]. As *R. slovaca* is known to be associated with lymphadenopathy and *R. raoultii* with similar disease [27],[44], our results suggest there is potential risk to humans by species of Rickettsiales detected in Xinjiang, which clearly warrants additional investigation.

Currently, the genus *Ehrlichia* contains five species and more than five unclassified genetic variants [9]. To date, only one study reports the presence of antibodies against *E. chaffeensis* in the ruminants from Xinjiang [23]. In this study, at least four species of *Ehrlichia* were discovered circulating in ticks in Xinjiang. Although the *rrs* tree could not provide resolution between newly discovered bacteria and previously characterized *Ehrlichia* species (Figure 1), the genetic separation is more obvious in the *gltA* and *groEL* genes, where the *Ehrlichia* sequences were clearly divided into four lineages. Remarkably, the sequences (*Ehrlichia* sp. BL116-7 and *Ehrlichia* sp. BL116-8) recovered from ticks from Bole were quite distinct from any known *Ehrlichia* spp. Thus, our data suggest that there are novel clades of *Ehrlichia* in Xinjiang ticks.

The genus *Anaplasma* includes six species [9]. Through analysis of a short fragment of *rrs*, *A. phagocytophilum* in *H. asiaticum* and sheep were recently found in other parts of Xinjiang [24],[39]. In this study, the bacteria (*Anaplasma* sp.TC249-5, *Anaplasma* sp.TC248-1, and *Anaplasma* sp.TC251-9) detected in ticks from Tacheng were closely related to *A. ovis* carried by *Dermacentor* spp. and *Rhipicephalus* spp. ticks in the *rrs*, *gltA*, and *groEL* trees, with 99.8%, 99.2%, and 99.7% nucleotide similarity, respectively, thereby indicating the presence of *A. ovis* in ticks from Tacheng region. As for *Anaplasma* sp. BL126-13 and *Anaplasma* sp. TC250-2 recovered in ticks from Bole and Tacheng, respectively, a closer relationship with *A. bovis* (98.9% and 86.1%) was observed in both *rrs* and *groEL* trees, suggesting that *A. bovis* circulates in ticks in Bole and Tacheng regions. Finally, the bacterial sequences (*Anaplasma* sp.BL102-7, *Anaplasma* sp.BL099-6, and *Anaplasma* sp.BL099-11) recovered from ticks form a lineage distinct from any known *Anaplasma*, suggesting a novel species circulating in ticks in this region.

It is important to note that bacterial endosymbionts are known to be abundant in tick species, although many are considered to be harmless to humans [45]. Further research is needed to confirm whether the sequences detected in this research are indeed from novel Rickettsiales species, and whether these species are endosymbiotic or potentially pathogenic.

An interesting observation of this study is that the phylogenetic analysis of this sample of sequences suggests that Rickettsiales may have originated in aquatic environments, with five adaptive shifts from an aquatic to terrestrial habitat. It had previously been suggested that the common ancestor of Rickettsiales was free-living, and that the transition to an intracellular lifestyle occurred 525-775 million years ago [6]. Interestingly, the genome of *R. bellii* includes many genes that are characteristic of amoebal symbionts [46], and it was suggested that the ancestors of *Rickettsia* could have used amoebae (or related protozoa) as hosts, from which further adaptation to terrestrial organisms, including ticks, occurred. For bacteria of the family *Candidatus* Midichloriaceae, aquatic/environmental protists likely have served as evolutionary reservoirs, from which one or more lineages evolved with the capacity to infect metazoans [11],[14]. In sum, all these data support the notion that aquatic/environmental protists played an important role in the evolution of the Rickettsiales [8],[11],[14],[16].

Our analyses also revealed that, although there is clearly congruence between the bacteria and vector/host trees, a model of exclusive bacteria-vector co-divergence can be rejected, albeit marginally. Hence, the biodiversity of the Rickettsiales must also reflect, at least in part, the occurrence of cross-species transmission. Host associations encompass free-living extracellular, facultative intracellular, and obligate intracellular (endosymbiotic) species, with the latter often exhibiting reductive genome evolution [21]. All of these lifestyles are accompanied by infecting new host species. It is possible that co-divergence occurred in the early stage of Rickettsiales evolution. This is apparent in the phylogeny (Additional file 4: Figure S3), in which species sampled from protists formed basal lineages to all *Rickettsia*, and that *Neorickettsia* (Trematoda/aquatic insect-associated) formed a basal lineage to *Wolbachia*, *Anaplasma*, and *Ehrlichia*. Since some Rickettsiales bacteria are endosymbiotic, their vertical transmission style may, to some degree, provide a mechanistic basis to the occurrence of co-divergence. However, it is clear that more data are needed to determine the precise evolutionary association between these bacteria and their vectors.

Finally, the diversity of tick-associated Rickettsiales is particularly noteworthy because both *Anaplasma* and *Ehrlichia* genera are tick-specific. In addition, the family *Rickettsia* has significant diversity associated with ticks, and some species of the family *Candidatus* Midichloriaceae are also found in ticks. For these tick-borne groups, the bacteria could be directly transmitted from aquatic protists to ticks. Alternatively, ticks could acquire microbes from other terrestrial organisms through cross-species transmission. Nevertheless, distinguishing between these two pathways is beyond the scope of this study and requires data from bacteria characterized from a variety of other organisms.

## Conclusions

Our screen for Rickettsiales bacteria in two tick species of Xinjiang revealed nine species, of which some *Ehrlichia* and *Anaplasma* species were distinct from any known Rickettsiales. Our phylogenetic analyses indicated that both co-divergence and cross-species transmission were responsible for the current evolutionary diversity of the Rickettsiales.

## Methods

### Tick sampling

During May 2011, ticks were collected from the Bole and Tacheng regions of Xinjiang Uygur Autonomous Region, China (Additional file 1: Figure S1). Ticks were directly obtained from domestic animals and grassland. All ticks were first identified morphologically by light microscopy and then verified by analyzing molecular markers [25]. The identified ticks were pooled into groups of 8 to 20 according to species and geographic origin, and stored at -70°C for subsequent screening for Rickettsiales bacterial DNA.

### DNA extraction, PCR and Sequencing

After washing twice with phosphate-buffered saline, ticks from each pool (or a single tick) were homogenized with a mortar and pestle in 1 mL (or 0.5 mL for individual tick) of phosphate-buffered saline solution. After homogenization, the suspension was incubated at 4°C for 1 h and centrifuged at 2,500 *g* for 5 min. The upper fraction was collected. DNA was then extracted from individual tick or tick pools with the DNeasy Tissue Kit according to the manufacturer’s instructions, and then subjected to PCR for amplification of bacterial gene sequences and tick mitochondrial rRNA genes (Qiagen, Valencia, CA, USA).

Rickettsial DNA was detected using PCR using primers fD1 and rD1 [47], which amplify a partial fragment (1.5 K) of Rickettsiales *rrs*. A negative control (distilled water) instead of tick DNA template in the PCR master mix, as well as a positive control (DNA from *E. chaffeensis*) were included in each test. To amplify gene sequences from samples positive for bacterial DNA, primers were designed based on conserved regions of complete *rrs*, *gltA*, and *groEL* gene sequences from the Rickettsiales spp. To determine whether the three gene sequences amplified from a pool of ticks were derived from a single tick sample, de-pool screening experiments were conducted. The three genes were screened from samples of new individual ticks. Finally, tick mitochondrial 12S and 18S rRNA genes were amplified as described previously [25].

The PCR products were purified with the Agarose Gel DNA Purification kit (TaKaRa, Dalian, China) according to the manufacturer’s recommendations. Purified DNA fragments were cloned into the pMD19-T vector (TaKaRa, Dalian, China), with the vector subsequently transformed into JM109-competent cells. At least 20 clones from each positive tick pool were selected for sequencing. DNA sequencing was performed using Applied Biosystems 377 gene sequencers at Shanghai Sangon Biological Engineering Technology and Services Co., Ltd. (Shanghai, China).

### Sequence data and genetic analyses

DNA sequences of the three bacterial genes (Additional file 6: Table S3) were aligned using ClustalW (default parameters) implemented in the MEGA program, version 5.2 [48]. The following data sets were then used in the evolutionary analysis: (i) a 1,243 bp *rrs* alignment (N = 110 sequences); (ii) a 466 bp *gltA* gene alignment (N = 79); and (iii) a 720 bp *groEL* gene alignment (N = 80). The 18S rRNA genes of the vectors (approximately 1700 bp, Additional file 7: Table S4) were aligned by R-coffee [49] with reference to rRNA predicted secondary structures. Finally, the sequences recovered in this study were named according to their relatedness with known bacteria, geographic origins, and sample numbers.

### Phylogenetic analyses

Phylogenetic trees were estimated using the Maximum Likelihood (ML) method implemented in the PhyML program (version 3) [50]. The General Time Reversible (GTR) nucleotide substitution model with a gamma (Γ)-distribution model of among-site rate variation and a proportion of invariable sites (i.e. the GTR + Γ + I substitution model) was utilized. Phylogenetic trees were also inferred using the Bayesian method implemented in MrBayes v3.2 [51]. The same substitution model was employed as described above. When using MrBayes v3.2, three hot chains and one cold Markov chain Monte Carlo (MCMC) were used, with trees and parameters sampled every 100 generations. A 25% burn-in was enforced for all analyses. Estimated sample sizes >200 for every model and search parameter were considered as indicators of adequate sampling of posterior distributions.

To infer the direction of evolutionary change within the order Rickettsiales (Additional file 6: Table S3, Additional file 8: Table S5), we inferred a molecular clock (i.e. rooted) phylogenetic tree using the BEAST software package (version 1.7.5) [52] assuming the GTR + Γ + I substitution model. This analysis also utilized the Yule process coalescent model. The MCMC chain was run for 10^8^ generations to ensure convergence. Statistical support for individual nodes was reflected in posterior probability values. Using the rooting determined in the BEAST tree, we then employed ML [53] and Bayesian [54] methods implemented in the Mesquite package [55] to tentatively reconstruct the evolution of habitat among the Rickettsiales by treating “aquatic vs. terrestrial” habitats as discrete character states and mapping their occurrence onto the phylogenies.

### Analysis of co-divergence events

We tested the hypothesis of bacterial-host co-divergence using the ParaFit method [56] as implemented in the COPYCAT software package [57], which compares the patristic distance matrices derived from the bacteria and vector phylogenies. For this analysis we prepared three tick-only data sets including (i) tick-associated *Rickettsia*, (ii) *Anaplasma*, and (iii) *Ehrlichia*, as well as an overall data set including all Rickettsiales. The bacterial genetic distance matrices were derived from the *rrs* trees inferred by both BEAST and ML methods, while the vector genetic distance matrices were derived from the 18S rRNA gene trees generated using BEAST as described above. Significance testing was based of 9,999 randomizations of the association matrices. Additionally, to illustrate the association between bacteria (Additional file 6: Table S3, Additional file 8: Table S5) and their vectors, a tanglegram was generated by matching each bacterial species (or group) to their associated vectors using TreeMap 3.0 [58].

## Competing interests

The authors declare that they have no competing interests.

## Authors’ contributions

YZZ conceived the research project; YZZ, YJK, XND, MHC, YX, WMF, YJG, and BP collected the samples, GYZ, YJK, and XPC performed research; YJK, GYZ, and MS analyzed the data; YZZ, YJK, MS, ECH, JJG, and SJD wrote the manuscript. All authors read and approved the final manuscript.

## Additional files

## Supplementary Material

Additional file 1: Figure S1. Map of sampling locations in Xinjiang province, China. The Bole and Tacheng regions are labeled by the red dots. Click here for file

Additional file 2: Table S1. Information of the sequences amplified in the ticks of Xinjiang. Click here for file

Additional file 3: Figure S2. Detailed ML phylogenetic trees based on the sequences of Rickettsiales rrs (a, d, g) , gltA (b, e, h), and groEL (c, f, i) genes. The numbers at each branch indicate bootstrap values. Click here for file

Additional file 4: Figure S3. Tanglegram of Rickettsiales bacteria and their hosts. The bacterial tree on the left panel of the figure was inferred based on rrs using BEAST and ML (PhyML) methods, while the vector tree on the right panel of the figure was inferred based on 18S rRNA sequences. Each bacterial species (or group) was linked to their associated vectors. In the bacterial tree different genera are distinguished by different colors. The BEAST tree is shown here. Click here for file

Additional file 5: Table S2. Results of the co-phylogeny analysis using ParaFit. Click here for file

Additional file 6: Table S3. Reference sequences used in this study. Click here for file

Additional file 7: Table S4. Sequences of the 18S rRNA gene of the vector species used in the phylogenetic analysis. Click here for file

Additional file 8: Table S5. Rickettsiales *rrs* (16S rRNA gene) sequences used in some analyses. Click here for file
