# Supplementary figures and images for: Extensive diversity of Rickettsiales bacteria in two species of ticks from China and the evolution of the Rickettsiales
Source: BMC Evol Biol. 2014 Jul 30;14:167. doi: 10.1186/s12862-014-0167-2 (PMC4236549; doi:10.1186/s12862-014-0167-2)

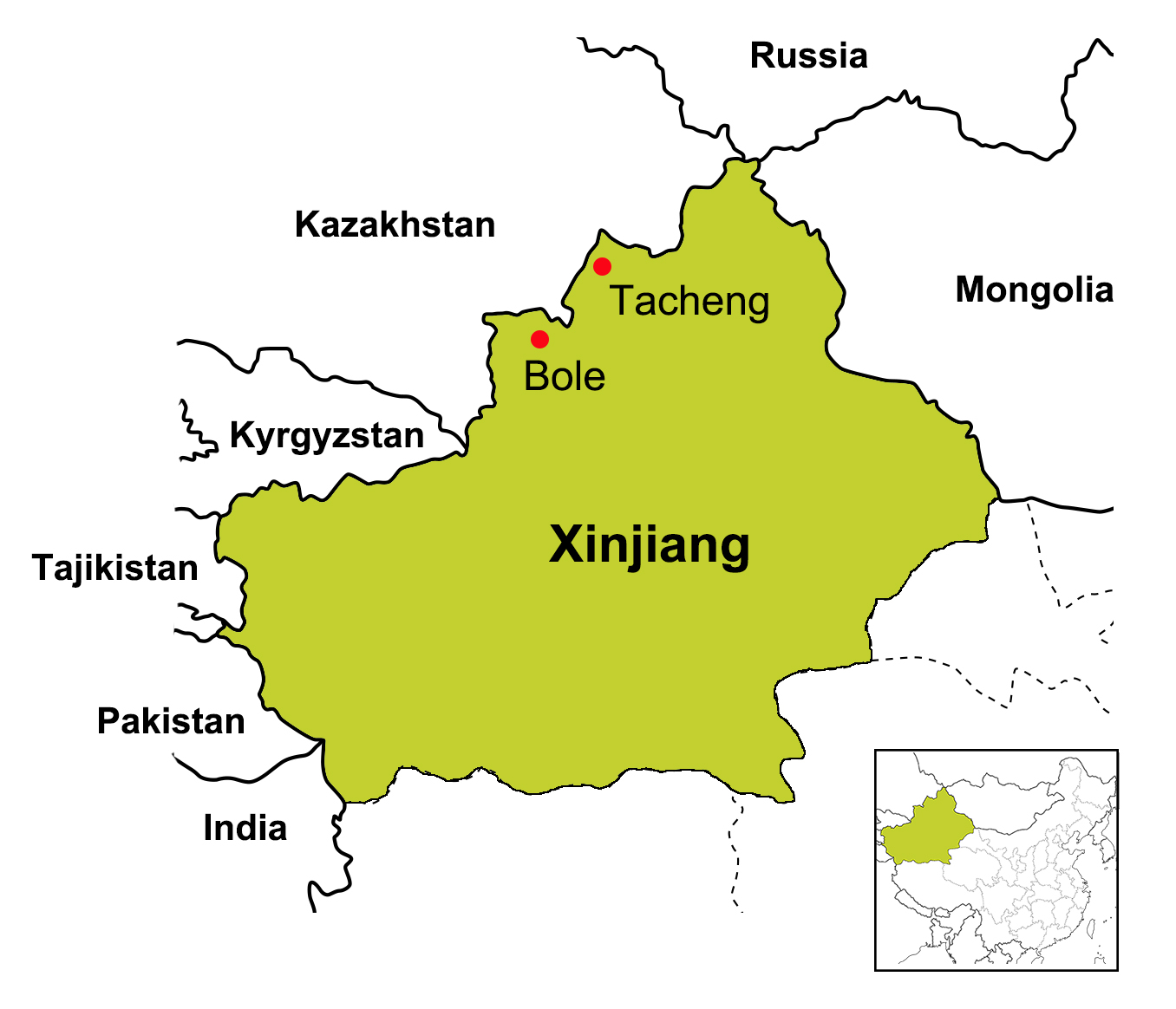

Supplement: Additional file 1: Figure S1. — Map of sampling locations in Xinjiang province, China. The Bole and Tacheng regions are labeled by the red dots. [file s12862-014-0167-2-S1.jpeg]

genus *Rickettsia*

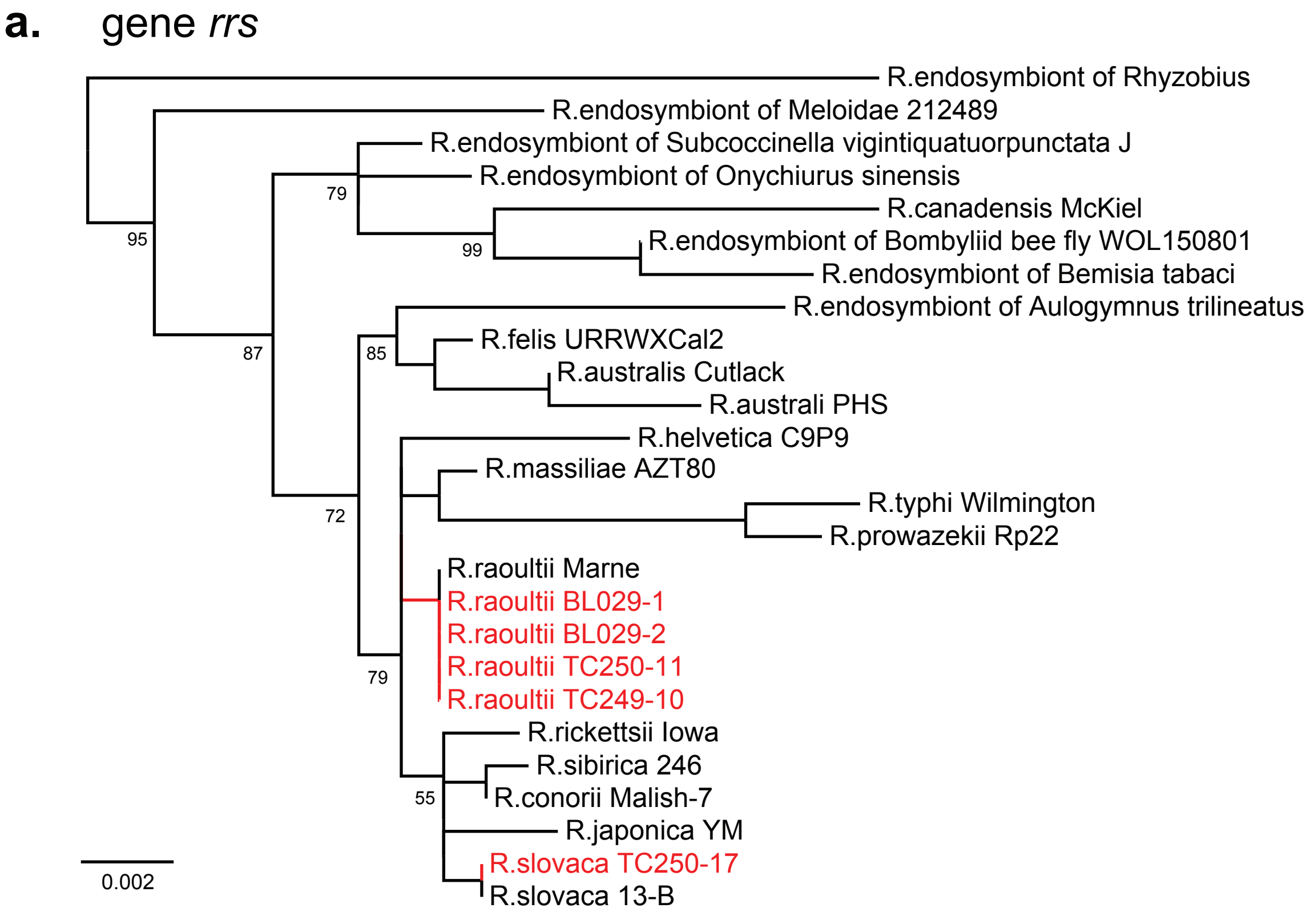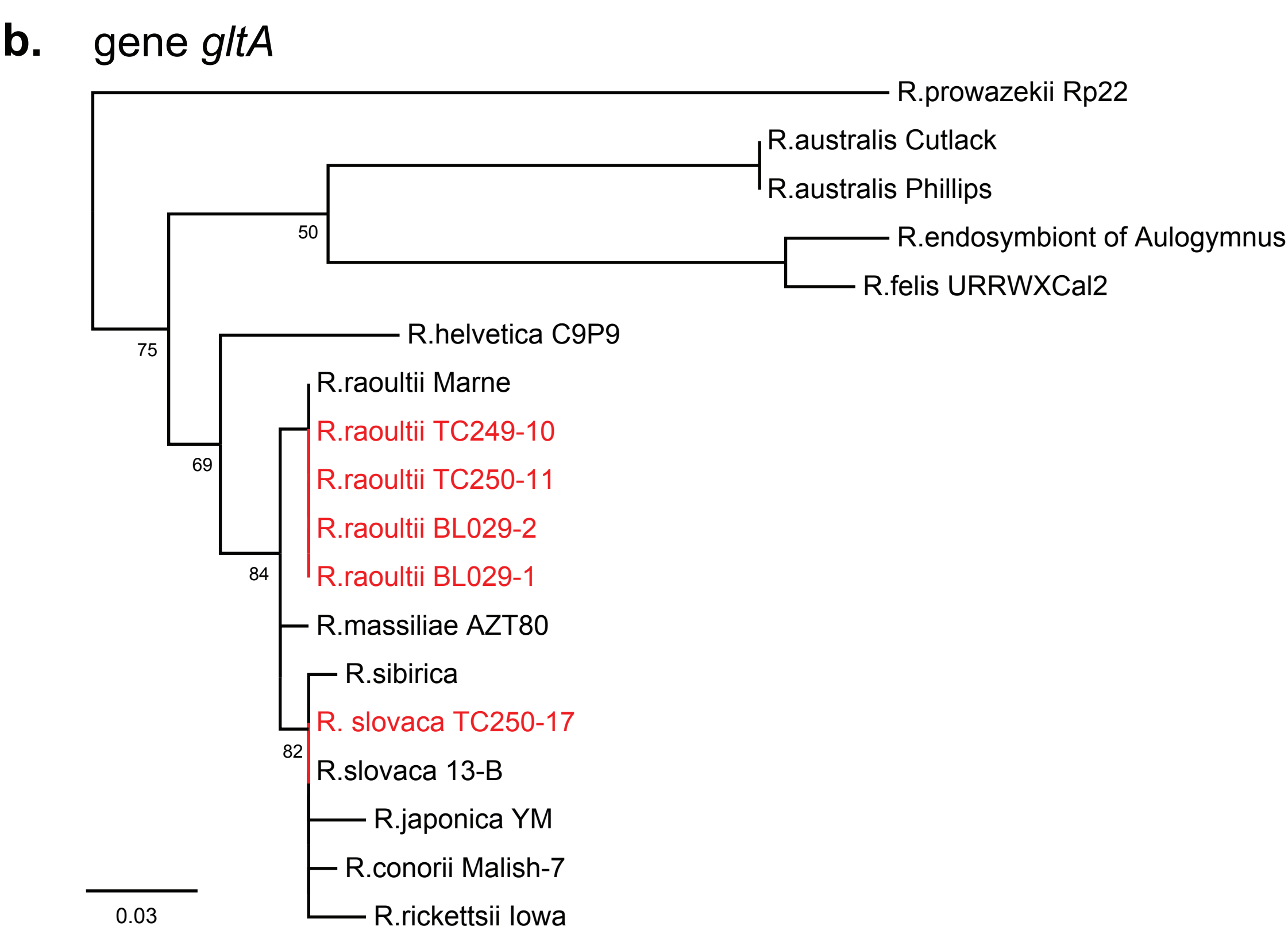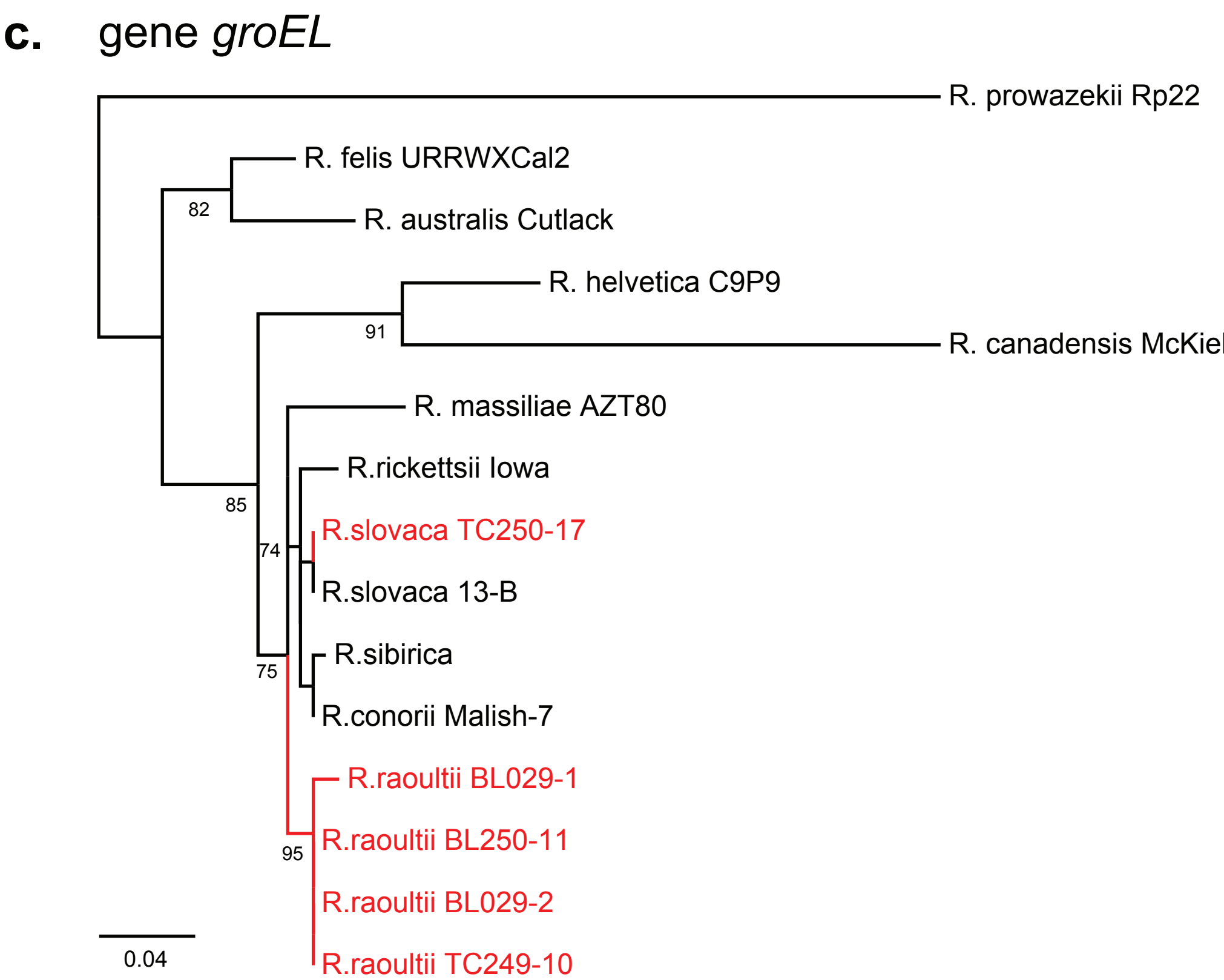

genus *Ehrlichia*

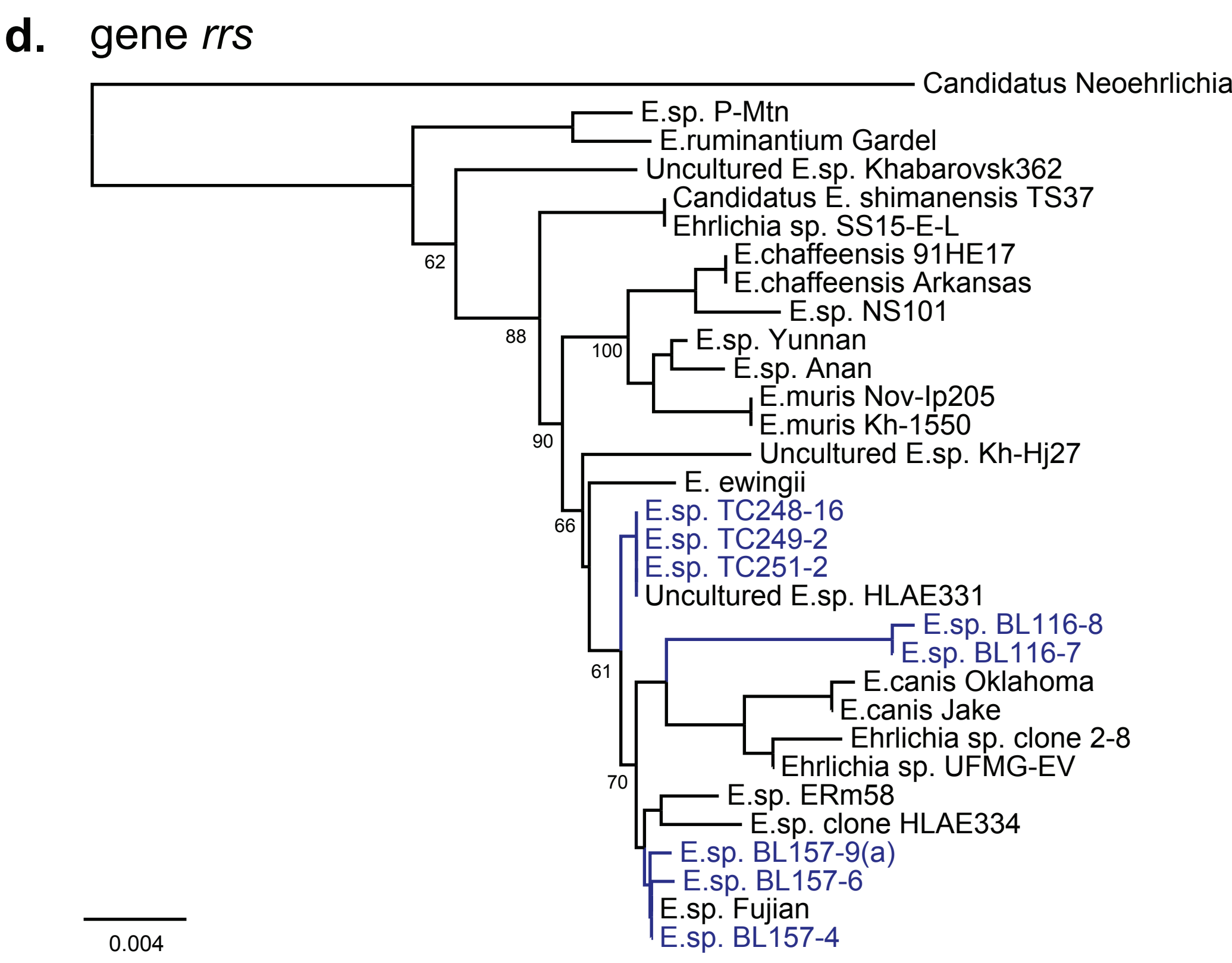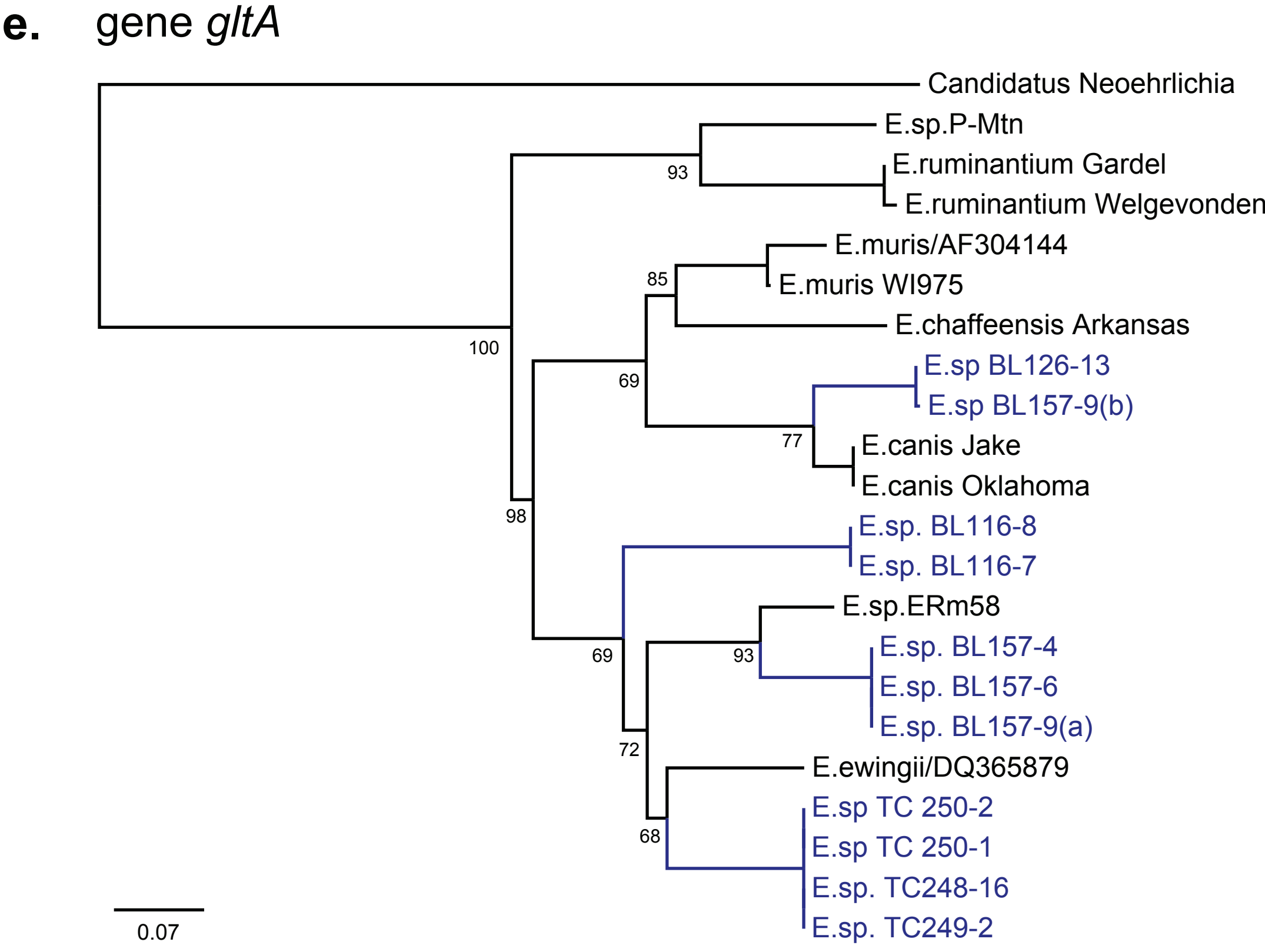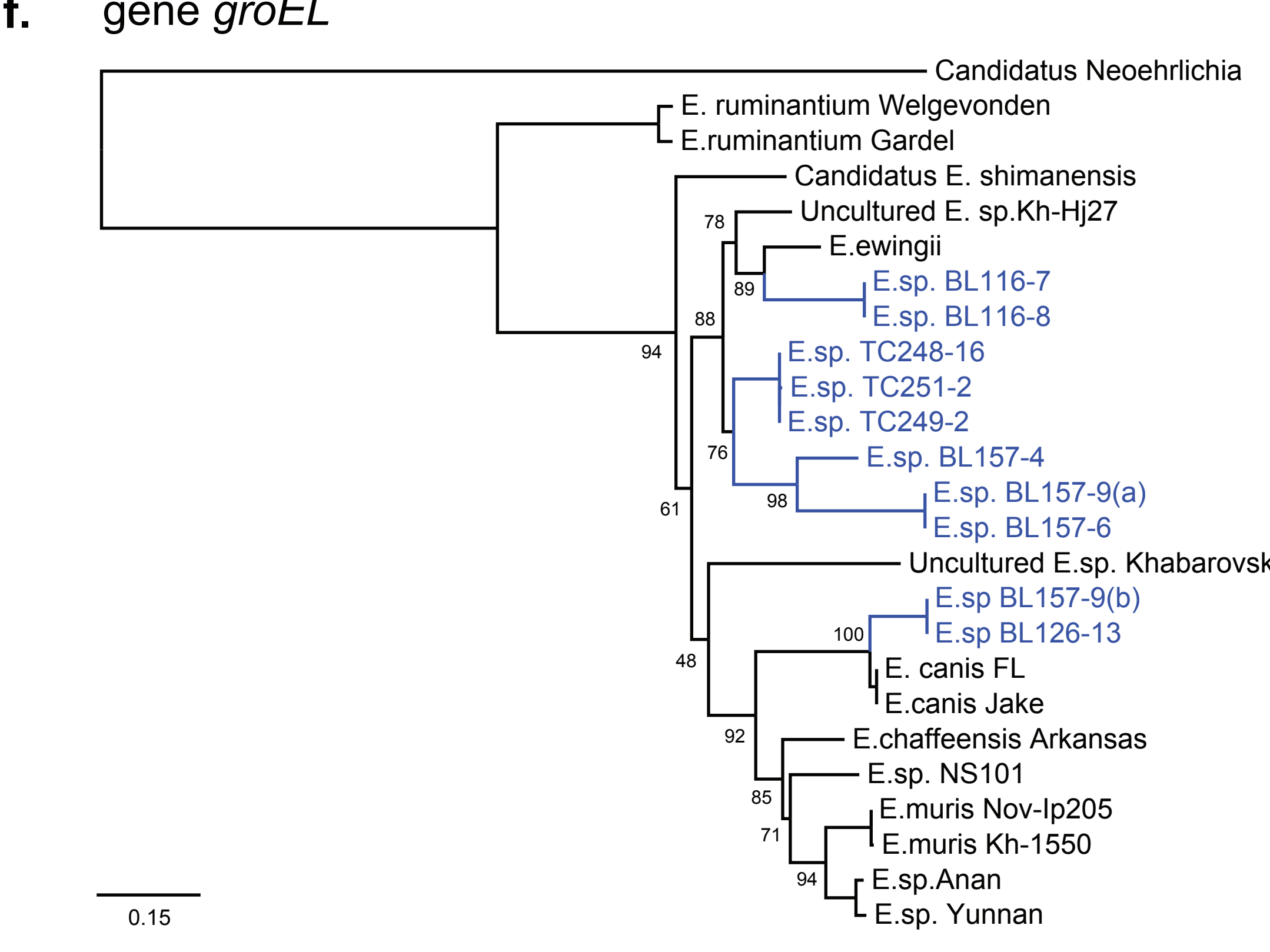

genus *Anaplasma*

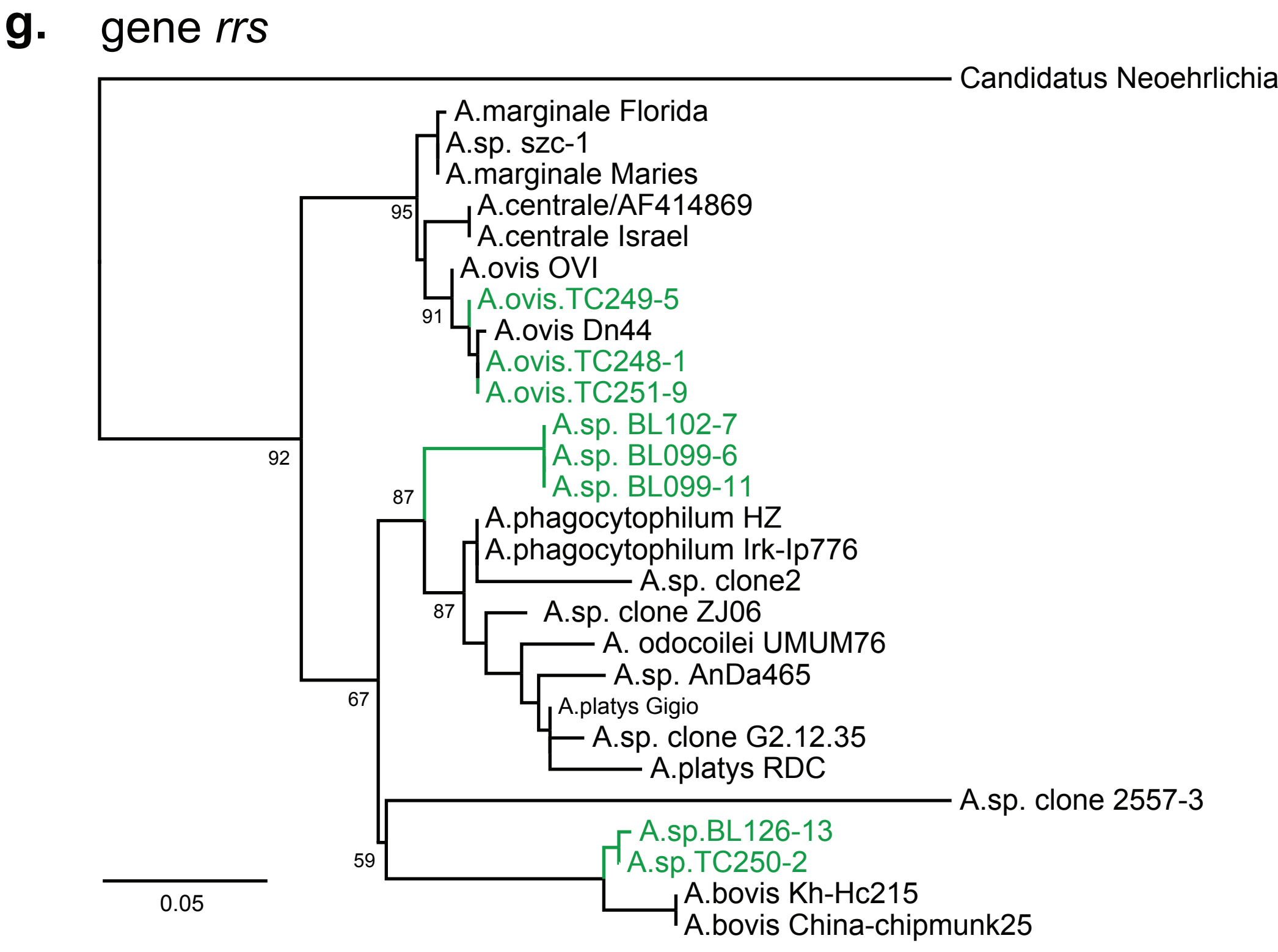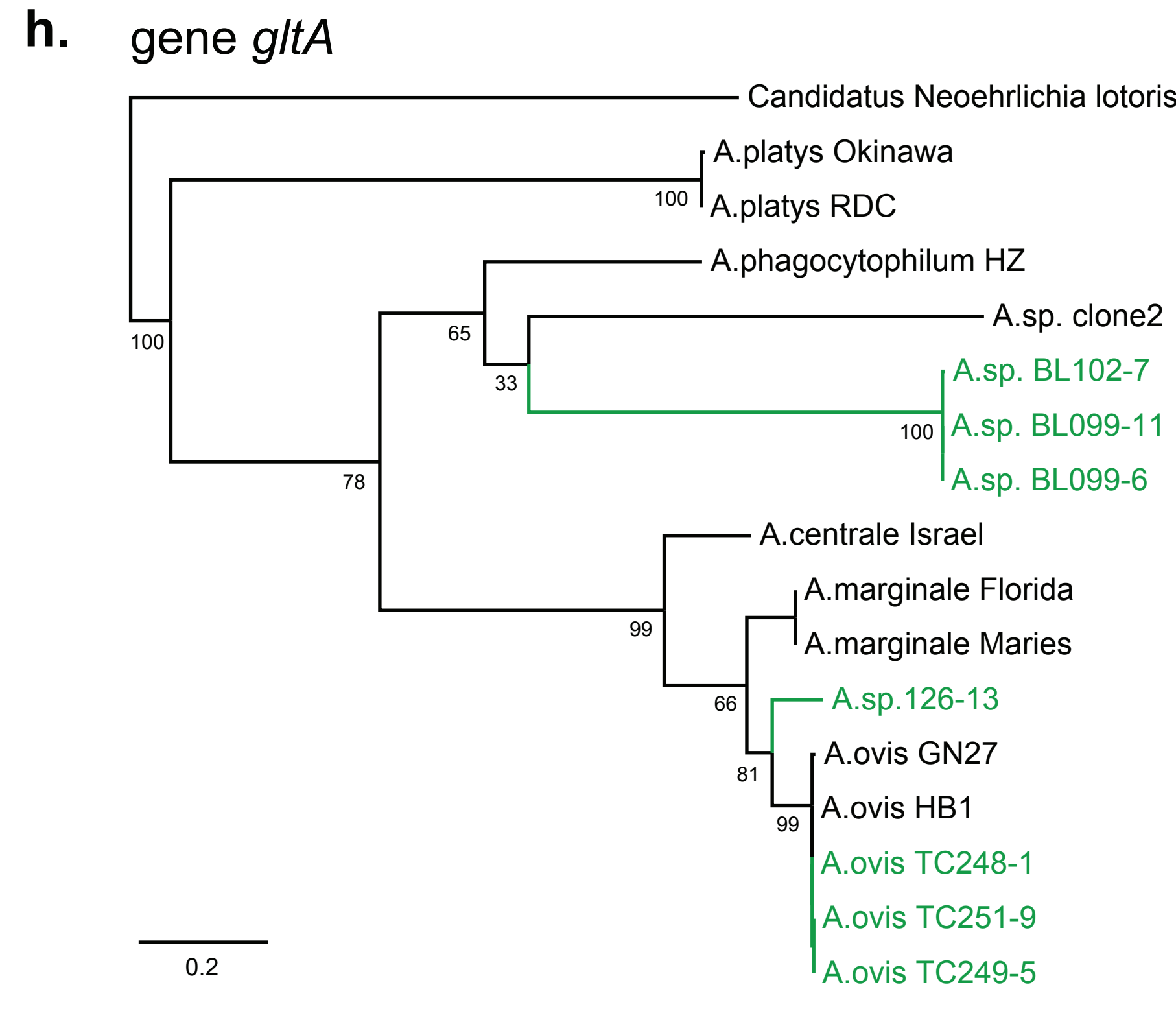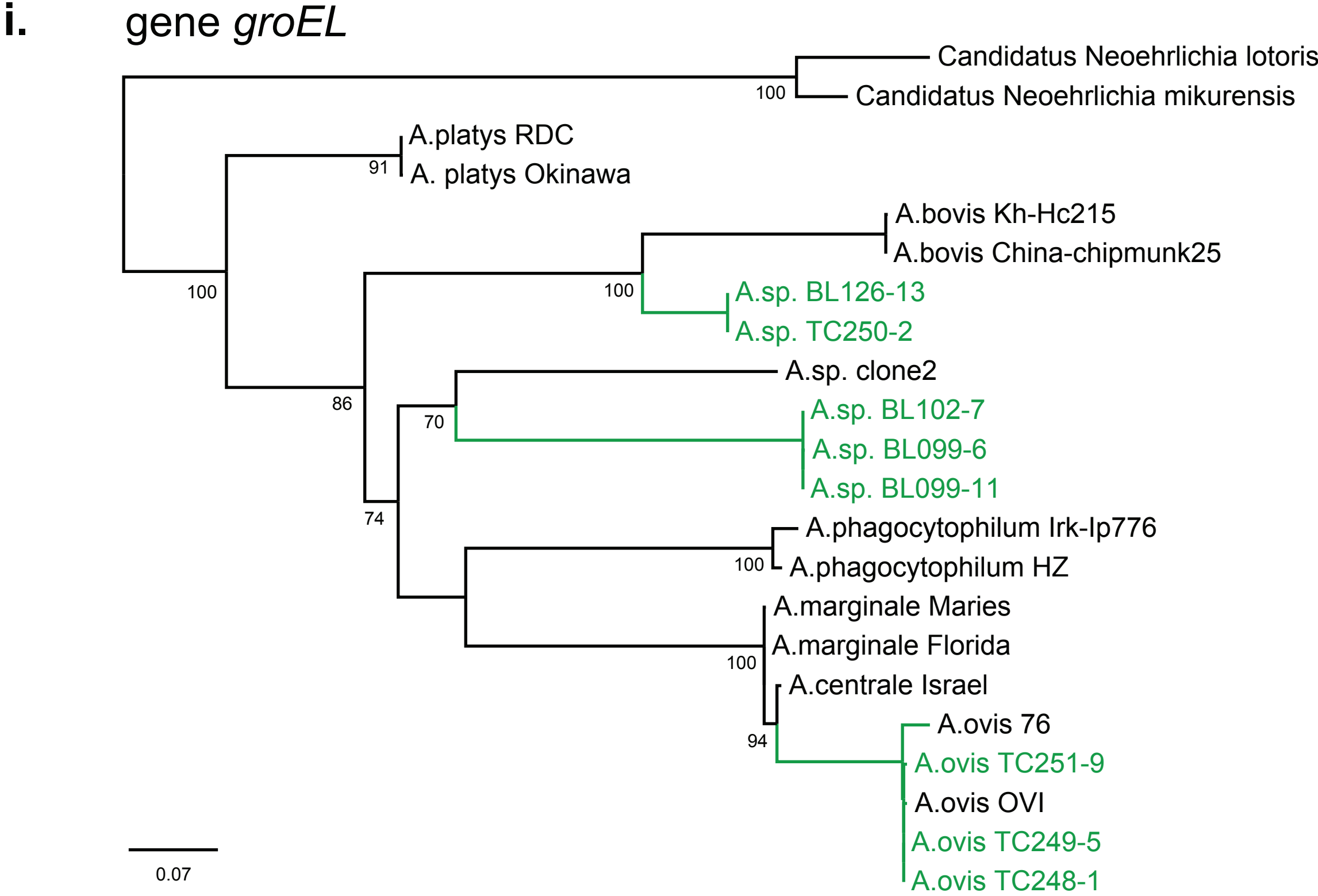

Supplement: Additional file 3: Figure S2. — Detailed ML phylogenetic trees based on the sequences of Rickettsiales rrs (a, d, g) , gltA (b, e, h), and groEL (c, f, i) genes. The numbers at each branch indicate bootstrap values. [file s12862-014-0167-2-S3.pdf]

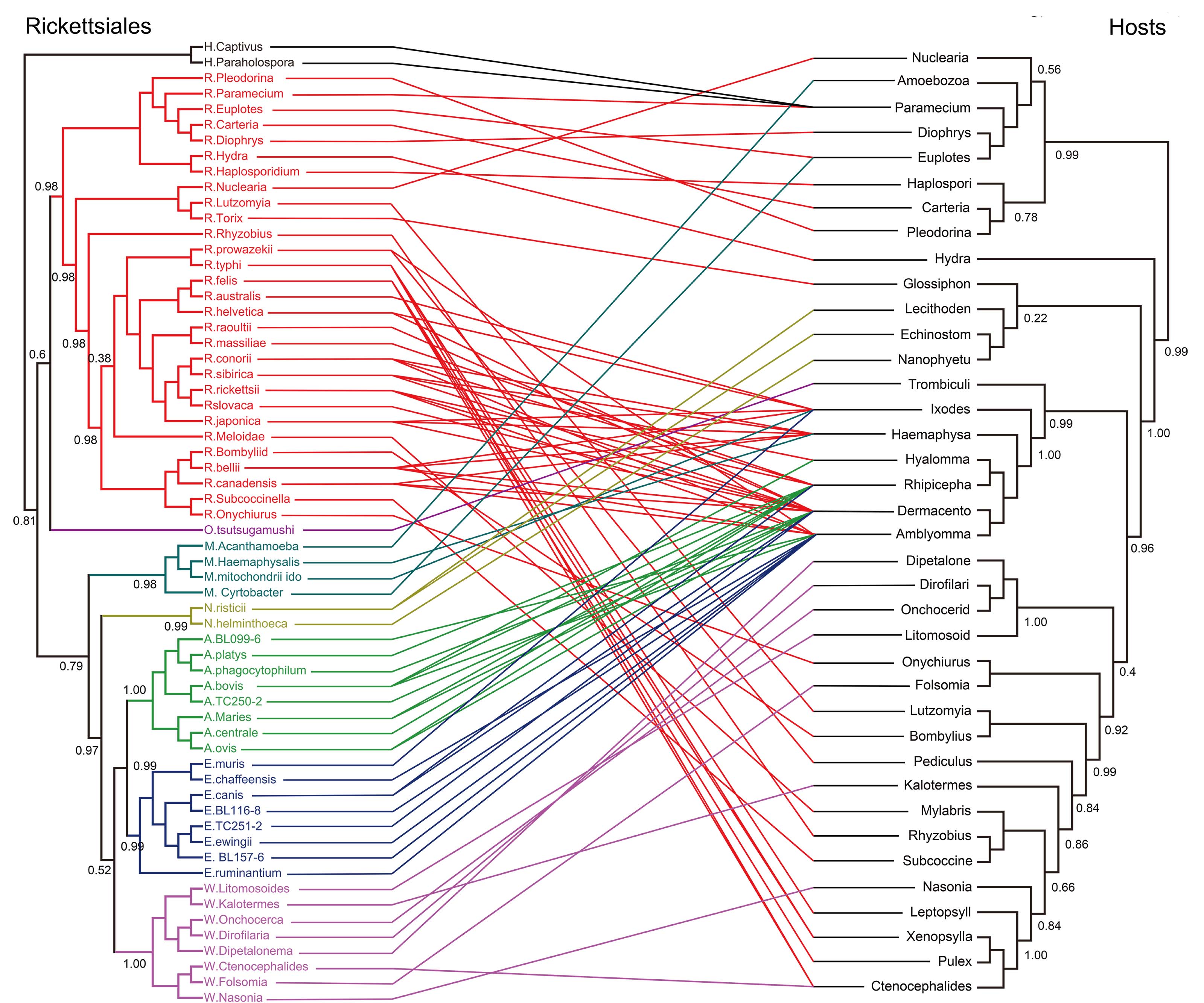

Supplement: Additional file 4: Figure S3. — Tanglegram of Rickettsiales bacteria and their hosts. The bacterial tree on the left panel of the figure was inferred based on rrs using BEAST and ML (PhyML) methods, while the vector tree on the right panel of the figure was inferred based on 18S rRNA sequences. Each bacterial species (or group) was linked to their associated vectors. In the bacterial tree different genera are distinguished by different colors. The BEAST tree is shown here. [file s12862-014-0167-2-S4.jpeg]
