# Supplementary material for: Extensive diversity of Rickettsiales bacteria in two species of ticks from China and the evolution of the Rickettsiales
Source: BMC Evol Biol. 2014 Jul 30;14:167. doi: 10.1186/s12862-014-0167-2 (PMC4236549; doi:10.1186/s12862-014-0167-2)
Supplement: Additional file 2: Table S1. — Information of the sequences amplified in the ticks of Xinjiang. [file s12862-014-0167-2-S2.doc]

Table S1. Information of the sequences amplified in the ticks of Xinjiang

| Location | Tick species | Sample no. | Sequences amplified (bp) / GenBank accession no. | | |
| --- | --- | --- | --- | --- | --- |
|  |  |  | *rrs* | *gltA* | *groEL* |
| ***Anaplasma*** | | | | | |
| Bole | *H. asiaticum* | BL099-6 | 1423/ KJ410247 | 1011/KJ410280 | 1143/KJ410301 |
| Bole | *H. asiaticum* | BL099-11 | 1376/ KJ410248 | 765/KJ410279 | 938/KJ410300 |
| Bole | *H. asiaticum* | BL102-7 | 1373/ KJ410249 | 731/KJ410281 | 961/KJ410302 |
| Bole | *H. asiaticum* | BL126-13 | 1281/ KJ410243 | 1024/KJ410282 | 1160/KJ410303 |
| Tacheng | *D. nuttalli* | TC250-2 | 1037/ KJ410242 | 0 | 1162/KJ410304 |
| Tacheng | *D. nuttalli* | TC248-1 | 1391 /KJ410244 | 855/KJ410283 | 783/KJ410297 |
| Tacheng | *D. nuttalli* | TC249-5 | 1382/ KJ410245 | 855/KJ410284 | 846/KJ410298 |
| Tacheng | *D. nuttalli* | TC251-9 | 1376/ KJ410246 | 855/KJ410285 | 785/KJ410299 |
| ***Ehrlichia*** | | | | | |
| Bole | *H. asiaticum* | BL126-13 | 604/ KJ410250 | 914/KJ410270 | 1295/KJ410292 |
| Bole | *H. asiaticum* | BL116-7 | 1387/ JX402603 | 865/KJ410268 | 1249/KJ410291 |
| Bole | *H. asiaticum* | BL116-8 | 1431/KJ410254 | 1079/KJ410269 | 1256/JX402610 |
| Bole | *H. asiaticum* | BL157-4 | 1427/KJ410255 | 874/KJ410271 | 1302/JX402612 |
| Bole | *H. asiaticum* | BL157-6 | 1326/KJ410256 | 874/KJ410272 | 1275/JX402613 |
| Bole | *H. asiaticum* | BL157-9(a) | 1348/KJ410257 | 620/KJ410273 | 1248/JX402611 |
| Bole | *H. asiaticum* | BL157-9(b) | 0 | 1086/KJ410274 | 885/KJ410293 |
| Tacheng | *D. nuttalli* | TC248-16 | 1394/KJ410251 | 1040/KJ410275 | 1288/KJ410294 |
| Tacheng | *D. nuttalli* | TC249-2 | 1388/KJ410252 | 845/KJ410276 | 1263/KJ410295 |
| Tacheng | *D. nuttalli* | TC251-2 | 1388/KJ410253 | 866/KJ410277 | 1262/KJ410296 |
| Tacheng | *D. nuttalli* | TC250-2 | 0 | 597/KJ410278 | 0 |
| ***Rickettsia*** | | | | | |
| Bole | *H. asiaticum* | BL029-1 | 1420/KJ410260 | 1011/KJ410263 | 1060/KJ410286 |
| Bole | *H. asiaticum* | BL029-2 | 1421/KJ410261 | 1040/KJ410264 | 1063/KJ410287 |
| Tacheng | *D. nuttalli* | TC249-10 | 1395/KJ410258 | 1166/KJ410266 | 1132/KJ410288 |
| Tacheng | *D. nuttalli* | TC250-11 | 1385/KJ410259 | 1063/KJ410265 | 1151/KJ410289 |
| Tacheng | *D. nuttalli* | TC250-17 | 1392/KJ410262 | 1000/KJ410267 | 1082/KJ410290 |
