# Supplementary material for: Extensive diversity of Rickettsiales bacteria in two species of ticks from China and the evolution of the Rickettsiales
Source: BMC Evol Biol. 2014 Jul 30;14:167. doi: 10.1186/s12862-014-0167-2 (PMC4236549; doi:10.1186/s12862-014-0167-2)
Supplement: Additional file 5: Table S2. — Results of the co-phylogeny analysis using ParaFit. [file s12862-014-0167-2-S5.doc]

Table S2. Results of the co-phylogeny analysis using ParaFit

| Tree | Rickettsiales bacterial species | ParaFitGlobal | *P* value |
| --- | --- | --- | --- |
| Rickettsiales | Species of order Rickettsiales hosted by various vectors | 4.17367 | 0.064 |
| *Anaplasma* | Species of genus *Anaplasma* hosted by various ticks | 0.00001 | 0.309 |
| *Ehrlichia* | Species of genus *Ehrlichia* hosted by various ticks | 0.00000 | 0.616 |
| *Rickettsia* | Species of genus *Rickettsia* hosted by various ticks | 0.00000 | 0.084 |
