# Supplementary material for: Extensive diversity of Rickettsiales bacteria in two species of ticks from China and the evolution of the Rickettsiales
Source: BMC Evol Biol. 2014 Jul 30;14:167. doi: 10.1186/s12862-014-0167-2 (PMC4236549; doi:10.1186/s12862-014-0167-2)
Supplement: Additional file 6: Table S3. — Reference sequences used in this study. [file s12862-014-0167-2-S6.doc]

Table S3. Reference sequences used in this study.

| Bacterial species | Strain | GenBank accession no. | | | | |
| --- | --- | --- | --- | --- | --- | --- |
|  |  | 16S | | gltA | | groEL |
| **Holosporaceae** |  |  | |  | |  |
| *Holospora curviuscula* | CCCS:MC-3 | KC164378 | |  | |  |
| *Holospora acuminata* | CCCS:KBN10-1 | KC164380 | |  | |  |
| *Candidatus* Gortzia infectiva | TS-a | HE797910 | |  | |  |
| *Candidatus* Paraholospora nucleivisitans |  | EU652696 | |  | |  |
|  |  |  | |  | |  |
| ***Candidatus* Midichloriaceae** |  |  | |  | |  |
| *Candidatus* Anadelfobacter veles | HS11/7 | FN552695 | |  | |  |
| *Candidatus* Defluviella procrastinata | 26 | HE978249 | |  | |  |
| *Candidatus* Lariskella arthropodarum | PhLaMyk | JQ726752 | |  | |  |
| *Candidatus* Midichloria mitochondrii | IricVA | NR_074492 | |  | |  |
|  |  |  | |  | |  |
| **Rickettsiaceae**  ***Orientia*** | | | | | | |
| *Orientia tsutsugamushi* | Boryong | NC_009488 | |  | | NC_009488 |
| *Orientia tsutsugamushi* | Ikeda | NC_010793 | |  | | NC_010793 |
|  |  |  | |  | |  |
| ***Rickettsia*** | | | | | | |
| Malla4.68 | AM945518 |  | |  | |  |
| AbFoot | AJ319724 |  | |  | |  |
| RS 8-Bact17 | AJ867656 |  | |  | |  |
| RB90b-112 | AM159487 |  | |  | |  |
|  | AB066351 |  | |  | |  |
| LA-1 | EU223247 |  | |  | |  |
| G | FJ609388 | FJ666753 | |  | |  |
| McKiel | CP000409 |  | | CP000409 | |  |
| 2678 |  | U59713 | |  | |  |
| Nagpur | JN204500 |  | |  | |  |
|  |  | DQ077708 | | EU435143 | |  |
| RML369-C | NC_007940 | NC_007940 | | NC_007940 | |  |
| PNG0405 |  | FJ666761 | |  | |  |
| WOL150801 | FJ609390 | FJ666755 | |  | |  |
|  | AY712949 |  | |  | |  |
| J | FJ609398 |  | |  | |  |
| 212489 | FJ609389 | FJ666754 | |  | |  |
| C9P9 | CM001467 | CM001467 | | CM001467 | |  |
| ATWASP107 | FJ609405 | FJ666769 | |  | |  |
| URRWXCal2 | NC_007109 | NC_007109 | | NC_007109 | |  |
| Cutlack | NC_017058 | NC_017058 | | NC_017058 | |  |
| Phillips | U12459 | U59718 | |  | |  |
| Marne | DQ365809 | DQ365803 | |  | |  |
| AZT80 | NC_016931 | NC_016931 | | NC_016931 | |  |
| Rp22 | CP001584 | CP001584 | | CP001584 | |  |
| Wilmington | NC_006142 | NC_006142 | | NC_006142 | |  |
| 246 | NZ_AABW01000001.1 | NZ_AABW01000001.1 | | NZ_AABW01000001.1 | |  |
| Malish-7 | NC003103 | NC003103 | | NC003103 | |  |
| 13-B | NC_016639 | NC_016639 | | NC_016639 | |  |
| YM | L36213 | U59724 | |  | |  |
| Iowa | NC_010263 | NC_010263 | | NC_010263 | |  |
|  |  |  |  | |  | |
| **Anaplasmataceae**  ***Neorickettsia*** | | | | | | |
| *Neorickettsia helminthoeca* |  | U12457 | | AF304149 | | AY050315 |
| *Neorickettsia risticii* | Illinois | NR_074389 | | AF304147 | | ERU96732 |
| *Neorickettsia sennetsu* | Miyayama | NC_007798 | | AF304148 | | U88092 |
|  |  |  | |  | |  |
| ***Wolbachia*** | | | | | | |
| *Wolbachia pipientis/Ctenocephalides felis* |  | AJ628416 | | AJ609650 | | AJ609659 |
| *Wolbachia* endosymbiont of *Kalotermes flavicollis* |  | Y11377 | | AJ609651 | | AJ609660 |
| *Wolbachia* endosymbiont of *Mansonella ozzardi* |  | AJ279034 | | AJ609647 | | AJ609657 |
| *Wolbachia* endosymbiont of *Onchocerca gibsoni* |  | AJ276499 | | AJ609639 | | AJ609652 |
| *Wolbachia* endosymbiont of *Dirofilaria immitis* |  | Z49261 | | AJ609641 | | AJ558023 |
| *Wolbachia* *pipientis*/*Dipetalonema gracile* |  | AJ548802 | | AJ609648 | | AJ609658 |
| *Wolbachia pipientis*/*Litomosoides brasiliensis* |  | AJ548799 | | AJ609646 | | AJ609655 |
| *Wolbachia* Endosymbiont/*Litomosoides sigmodontis* |  | AF069068 | | AJ609645 | | AF409113 |
| *Wolbachia* endosymbiont/*Nasonia vitripennis* |  | M84686 | | AY714782 | | AY714796 |
| *Wolbachia* endosymbiont of *Culex pipiens* |  | X61768 | | AY714785 | |  |
| *Wolbachia* endosymbiont of *Nasonia vitripennis* |  | M84688 | | AY714795 | |  |
| *Wolbachia* pipientis/*Folsomia candida* |  | AF179630 | | AJ609649 | |  |
|  |  |  | |  | |  |
| ***Ehrlichia*** | | | | | | |
| *Candidatus* Neoehrlichia lotoris | RAC413 | EF633744 | | EF633746 | | F633745 |
| *Candidatus* Neoehrlichia mikurensis | 2010HLJ936H | JQ359045 | |  | | JQ359062 |
| Uncultured *Ehrlichia* sp. | Khabarovsk_362 | FJ966352 | |  | | FJ966351 |
| *E. ruminantium* | Gardel | NC_006831 | | NC_006831 | | NC_006831 |
| *E. ruminantium* | Welgevonden |  | | NC_006832 | | NC_006832 |
| *Ehrlichia.* sp*.* | P-Mtn | DQ324367 | | EU272375 | |  |
| *Ehrlichia.* sp*.* | NS101 | AB454074 | |  | | AB454077 |
| *E. chaffeensis* | Arkansas | NC_007799 | | NC_007799 | | NC_007799 |
| *E. chaffeensis* | 91HE17 | U23503 | |  | |  |
| *E. muris* | KH1550 | GU358692 | |  | | GU358689 |
| *E. muris* | Nov-Ip205 | GU358691 | |  | | GU358686 |
| *E. muris* | WI975 |  | | HQ660497 | |  |
| *E. muris* |  |  | | AF304144 | |  |
| *Ehrlichia.* sp*.* | Anan | AB028319 | |  | | AB032711 |
| *Ehrlichia.* sp*.* | Yunnan | GU227701 | |  | | GU227700 |
| *E. shimanensis* | TS37 | AB074459 | |  | | AB074462 |
| *Ehrlichia.* sp*.* | Kh-Hj27 | FJ966350 | |  | | FJ966349 |
| *E. ewingii* |  | NR_044747 | | DQ365879 | | AF195273 |
| *Ehrlichia.* sp*.* | HLAE331 | GU075697 | |  | |  |
| *Ehrlichia.* sp*.* | Fujian | DQ324547 | |  | |  |
| *E. canis* | Jake | NC_007354 | | NC_007354 | | NC_007354 |
| *E. canis* | Oklahoma | M73221 | | AF304143 | |  |
| *E. canis* | FL |  | |  | | U96731 |
| *Ehrlichia.* sp*.* | ERm58 | AF311967 | | AF311965 | |  |
|  |  |  | |  | |  |
| ***Anaplasma*** | | | | | | |
| *A. centrale* |  | AF414869 | |  | |  |
| *A. centrale* | Israel | NC_013532 | | NC_013532 | | NC_013532 |
| *A. ovis* | OVI | AF414870 | |  | | AF441131 |
| *A. ovis* | Tuva-Dn4420 | KC484562 | |  | |  |
| *A. ovis* | HB1 |  | | JX559690 | |  |
| *A. ovis* | GN27 |  | | JX559689 | |  |
| *A. ovis* | 76 |  | |  | | FJ460441 |
| *Anaplasma* sp*.* | szc-1 | FJ389579 | |  | |  |
| *A. marginale* | Florida | NC_012026 | | NC_012026 | | NC_012026 |
| *A. marginale* | Maries | NC_004842 | | NC_004842 | | NC_004842 |
| *A. phagocytophilum* | HZ | NC_007797 | | NC_007797 | | NC_007797 |
| *A. phagocytophilum* | Irk-Ip776 | HM366585 | |  | |  |
| *A. platys* | RDC | AF478131 | | AF478130 | | AF478129 |
| *A. platys* | Gigio | EU439943 | |  | | HM366573 |
| *A. platys* | Okinawa |  | | AY077620 | |  |
| *Anaplasma sp.* | clone_2 | JN055358 | | JN055362 | | JN055360 |
| *A. bovis* | Kh-Hc215 | JX092094 | |  | | JX092095 |
| *A. bovis* | China-chipmunk25 | JX092096 | |  | | JX092097 |
