# Supplementary material for: Extensive diversity of Rickettsiales bacteria in two species of ticks from China and the evolution of the Rickettsiales
Source: BMC Evol Biol. 2014 Jul 30;14:167. doi: 10.1186/s12862-014-0167-2 (PMC4236549; doi:10.1186/s12862-014-0167-2)
Supplement: Additional file 7: Table S4. — Sequences of the 18S rRNA gene of the vector species used in the phylogenetic analysis. [file s12862-014-0167-2-S7.doc]

Table S4. Sequences of the 18S rRNA gene of the vector species used in the phylogenetic analysis

| Vector species* | GenBank accession no. |
| --- | --- |
| *Nuclearia pattersoni* | AY364635 |
| *Haplosporidium montforti* | DQ219484 |
| *Paramecium caudatum* | HE662765 |
| *Diophrys appendiculata* | AY004773 |
| *Euplotes octocarinatus* | AJ310489 |
| *Carteria cerasiformis* | AB688624 |
| *Pleodorina japonica* | AB688627 |
| *Hydra oligactis* | JN594052 |
| *Lecithodendriidae* | EU019964 |
| *Echinostoma caproni* | L06567 |
| *Nanophyetus salminicola* | AY222138 |
| *Glossiphonia verrucata* | AY962432 |
| *Trombiculidae cf. Hoffmaniella* | HM070354 |
| *Dipetalonema* | DQ531723 |
| *Litomosoides sigmodontis* | AF227233 |
| *Dirofilaria immitis* | AF036638 |
| *Onchoceridae* | DQ103704 |
| *Onychiurus yodai* | AY037171 |
| *Folsomia candida* | KC236240 |
| *Lutzomyia nevesi* | AB638310 |
| *Bombylius* | KC177290 |
| *Pediculus humanus* | AY077775 |
| *Meloidae* | JN619260 |
| *Rhyzobius litura* | EF512321 |
| *Subcoccinella* | AY748149 |
| *Nasonia vitripennis* | GQ410677 |
| *Leptopsylla segnis* | DQ298442 |
| *Ctenocephalides felis* | KC177274 |
| *Pulex irritans* | AF423915 |
| *Xenopsylla cheopis* | DQ298440 |
| *Kalotermes flavicollis* | FJ806330 |
| *Ixodes persulcatus* | AY274888 |
| *Amblyomma americanum* | AF291874 |
| *Dermacentor andersoni* | L76340 |
| *Rhipicephalus sanguineus* | JX987497 |
| *Hyalomma lusitanicum* | Z74482 |
| *Haemaphysalis flava* | JX573120 |

* Sequences from members of the same genus were substituted for species that lack 18S rRNA sequences in GenBank.
