# Supplementary material for: Extensive diversity of Rickettsiales bacteria in two species of ticks from China and the evolution of the Rickettsiales
Source: BMC Evol Biol. 2014 Jul 30;14:167. doi: 10.1186/s12862-014-0167-2 (PMC4236549; doi:10.1186/s12862-014-0167-2)
Supplement: Additional file 8: Table S5. — Rickettsiales rrs (16S rRNA gene) sequences used in some analyses. [file s12862-014-0167-2-S8.doc]

Table S5. Rickettsiales *rrs* (16S rRNA gene) sequences used in some analyses.

| Bacterial species | Strain | GenBank  accession no. |
| --- | --- | --- |
| **Bacteria used in the Tanglegram** |  |  |
| Endosymbiont of *Euplotes octocarinatus* | FL(12)-VI | FR823004 |
| Bacterial symbiont of *Diophrys* sp. |  | AJ630204 |
| Endosymbiont of *Paramecium caudatum* | SH42 | FR822997 |
| Rickettsiaceae endosymbiont of *Carteria cerasiformis* | NIES-425 | AB688628 |
| Rickettsiaceae endosymbiont of *Pleodorina japonica* | NIES-577 | AB688629 |
| Uncultured *Rickettsia* sp. / *Hydra* | EHFS1_S06b | EU071486 |
| *Rickettsia* endosymbiont of *Nuclearia pattersoni* |  | AY364636 |
|  |  |  |
| **Bacteria used in the ancestral reconstruction** |  |  |
| *Candidatus* Cryptoprodotis polytropus |  | FM201293 |
| Uncultured Rickettsiales bacterium/*Hydra vulgaris* | Hv | EF667921 |
| Uncultured Rickettsiales bacterium/*Mnemiopsis leidyi* | Mn6_5 | GQ926866 |
| Uncultured deep-sea bacterium | Ucm1520 | AM997318 |
| Uncultured bacterium/Dog flea | Oh3123O11E |  |
| Uncultured Rickettsiales bacterium/*Apostichopus japonicus* | B-09-239 | AB550452 |
